# Identification of intratumoral bacteria correlated with hepatitis B virus (HBV) levels: a prognostic indicator for patient outcomes in hepatocellular carcinoma patients

**DOI:** 10.1128/spectrum.01188-25

**Published:** 2025-10-23

**Authors:** Yuan Dang, Jingyun Huang, Xing Peng, Yingchao Wang, Jianmin Wang

**Affiliations:** 1 Innovation Center for Cancer Research, Clinical Oncology School of Fujian Medical University, Fujian Cancer Hospital 66552 , Fuzhou, Fujian, China; 2 Fujian Key Laboratory of Advanced Technology for Cancer Screening and Early Diagnosis, Clinical Oncology School of Fujian Medical University, Fujian Cancer Hospital 66552 , Fuzhou, China; 3 College of Computer and Data Science, Fuzhou University 12423, Fuzhou, China; 4 The United Innovation of Mengchao Hepatobiliary Technology Key Laboratory of Fujian Province, Mengchao Hepatobiliary Hospital of Fujian Medical University 477092, Fuzhou, China; Children's National Hospital, George Washington University, Washington, DC, USA

**Keywords:** hepatocellular carcinoma, hepatitis B, viral load, tumor tissue microbiome, prognosis

## Abstract

Hepatocellular carcinoma (HCC) is a prevalent liver cancer associated with global health. The tumor microenvironment’s (TME) microbial composition significantly influences HCC’s development and prognosis. This study analyzed bacterial 16S rRNA genes from liver cancer patients’ tumor, adjacent tumor, and normal tissues, revealing distinct microbiome profiles between tumor and normal tissues. HCC microbiota was more abundant, with *Bacteroides*, *Ochrobactrum*, *Akkermansia*, and *Lactobacillus* as the most prevalent genera. Hepatitis B virus (HBV)-positive (HBV+) and HBV-negative (HBV−) HCC tissues showed different microbial network patterns, with *Bacteroides* enrichment in HBV+ tissues being associated with HCC prognosis. HBV is associated with clinicopathological features and serves as an independent prognostic factor in HCC. The study underscores the microbiota’s complexity in HCC and the potential of HBV as a prognostic biomarker post-surgery. This study provides critical insights into the relationship between the microbiota within the TME and HCC, a leading cause of cancer-related mortality. By identifying distinct microbiome profiles in HCC patients, particularly the enrichment of *Bacteroides* in HBV-positive tissues, our research not only uncovers the complexity of microbial interactions in liver cancer but also highlights the potential of using HBV status as a prognostic biomarker. This could significantly inform personalized treatment strategies and improve clinical outcomes for HCC patients, emphasizing the relevance of microbiome-based diagnostics and therapies in oncology.

**IMPORTANCE:**

In our study of the tumor microenvironment (TME) in hepatocellular carcinoma (HCC), we used DNA sequencing of bacterial 16S rRNA genes to analyze microbial compositions in tumor, adjacent tumor, and normal tissues from 213 liver cancer patients. Fluorescence *in situ* hybridization confirmed the presence of microbiota within tumors. Our results showed significant differences in microbiome profiles between tumor and normal tissues, with increased abundance of *Bacteroides*, *Ochrobactrum*, *Akkermansia*, and *Lactobacillus* in the HCC TME. Hepatitis B virus (HBV) status further stratified these differences, with *Bacteroides* significantly enriched in HBV-positive tissues and correlating with patient prognosis. Additionally, *Bacteroides* and *Akkermansia* showed interdependent population changes. Clinicopathological features, such as tumor size, were associated with HBV status, identifying HBV infection as an independent prognostic factor. These findings highlight the HCC microbiota’s complexity and suggest HBV status as a potential prognostic biomarker, opening avenues for personalized therapeutic strategies.

## INTRODUCTION

Hepatocellular carcinoma (HCC), intrahepatic cholangiocarcinoma (ICC), and hybrid HCC-ICC are the three main pathological forms of primary hepatocellular carcinoma, a malignant tumor arising from hepatocytes or intrahepatic bile duct epithelial cells (1, 2). With HCC accounting for more than 90% of cases, there are several significant differences between the three types with regard to causation, biological behavior, histological morphology, treatment methods, and prognosis (3). According to the most recent international cancer data released by the International Agency for Research on Cancer of the World Health Organization as of December 2020, primary liver cancer ranks seventh among malignant tumors in terms of incidence rate, with up to 906,000 new cases, and third in terms of mortality, with 830,000 cases total (2, 4). Among all malignant tumors, primary liver cancer ranks fifth in incidence in China each year, accounting for up to 410,000 new cases. Globally, there have been 995,000 primary liver cancer cases annually on average over the last 5 years. Of these, 73.6%, or 732,000 cases, were found in Asia, while 42.5%, or 423,000 instances, occurred in China. One of the main risk factors for primary HCC is cirrhosis, which can arise from a number of different causes (2). The etiologic composition of HCC in China is dominated by chronic hepatitis B virus (HBV) infection, accounting for approximately 86% of cases.

Trillions of microorganisms reside in the human body (5), some of which are involved in physiological reactions that are either carcinogenic or noncarcinogenic (6). Tumor tissues contain a variety of microorganisms, such as viruses, bacteria, fungi, and mycoplasmas. Cancer cells and immune cells that have invaded can contain microbial remnants, including DNA, RNA, peptides, and parts of the cell wall. Certain metabolites of microorganisms, such as inosine and fatty acids, can accumulate in tumors and attach to immune and cancer cell receptors. This new milieu, which differs from the frequently discussed tumor microenvironment (TME), can be referred to as the “tumor microbial microenvironment” and is crucial to carcinogenesis, progression, metastasis, and the immune response (7).

The tissue-enriched flora and microbiota observed in tumors, in addition to the gut flora, may also be linked to carcinogenesis. An increasing body of research suggests that distinct intratumoral bacterial flora are present in human malignancies, including breast, lung, ovarian, pancreatic, melanoma, bone, and brain tumors (8). Surprisingly, intratumoral bacteria are intracellular and are present in both immune and cancer cells in the majority of cancer types (8). There are two main categories of microorganisms observed in the tumor microenvironment of hepatocellular carcinoma: bacteria and viruses. One of the main risk factors for liver cancers, such as HCC and cirrhosis, is HBV infection. Research has demonstrated that HBV infection can change the dynamics of the gut microbiota (9). Transmission electron microscopy was used to identify intracellular bacteria in intrahepatic cholangiocarcinoma (a type of hepatocellular carcinoma) and the tissues surrounding it, but no additional detailed characterization of these bacteria was provided (10). *Enterococcus faecalis* was found in hepatocellular carcinoma samples, indicating that this bacterium may be important for the development of hepatocarcinogenesis (11).

The microbiological composition of liver cancer tumors remains unclear. Multiple bacterial DNAs have been found in the liver tissues of patients with nonalcoholic fatty liver disease, according to recent investigations (12). However, we are unsure of the survival rate and location of these bacteria within tumors. In addition, attention should be given to the dysbiosis of the HCC microbiota, the taxa of HCC that are unique to certain disease states, and the correlation between the bacterial burden and the clinicopathological traits of HCC patients. In particular, the connection between microorganisms and HBV has been investigated. Hepatic disorders, including HCC, are mostly mediated by the gut microbiota and liver in a reciprocal relationship (13). There is mounting evidence that the gut microbiome influences the onset and course of HCC. Furthermore, the gut microbiota may be used as a predictive and early diagnostic marker for HCC therapy. As a result, altering the gut microbiota may constitute a novel therapeutic or preventative method for treating HCC (14). The gut microbiota may be a useful biomarker for predicting HCC recurrence at an early stage, according to recent research, and the gut microbiota-tumor metabolite axis may provide insight into the possible mechanism by which the gut microbiota promotes early recurrence of HCC (15). The findings regarding the heterogeneity of bacterial butyric acid production in HBV-negative (HBV−) and HBV-positive (HBV+) HCC patients further demonstrate the potential role of HBV in causing changes in the gut flora. HBV+ and HBV− HCC are associated with different bacteria and display abnormal microbial community ecological networks (16).

In this study, we investigated the differences in the microbiota in the tumor, adjacent tumor, and normal areas of patients with primary liver cancer and conducted an in-depth analysis of HCC patients. Based on patients with a high viral load (HBV DNA level ≥2,000 IU/mL, HBV+), and low viral load (HBV DNA <2,000 IU/mL, HBV−) (17), we studied differences in the tumor microbiota associated with different viral loads in HBV-infected HCC patients. By analyzing the influence of different microorganisms in the tumors of HBV+ and HBV− patients on the prognosis of HCC patients, the marker microorganisms of HBV infection were identified, and the effects of viruses on microorganisms were investigated to effectively prevent HBV infection in HCC patients.

## MATERIALS AND METHODS

### Sample collection

A total of 213 patients with primary liver cancer were recruited from Mengchao Hepatobiliary Hospital. The median follow-up duration was 34.6 months. Inclusion criteria were as follows: (i) histologically confirmed primary hepatocellular carcinoma and (ii) availability of clinical data at the time of diagnosis. Patients who received preoperative care or cancer treatment, as well as those with viral infections other than HBV, were excluded. Ultimately, 213 patients met the inclusion and exclusion criteria. Tumor, adjacent, and normal tissues were collected from these 213 patients, yielding a total of 639 tissue samples. All samples were collected under sterile conditions in the operating room and subsequently cryopreserved at −80°C within 30 minutes after collection.

### DNA extraction and 16S rDNA sequencing

DNA was used to extract genomic DNA from the tumor, adjacent, and normal tissue samples. Amplification was then carried out, and the integrity of the PCR products was evaluated by electrophoresis on a 1.8% agarose gel (Beijing Bomei Fuxin Technology Co., Ltd.). A NanoDrop 2000 (Thermo Scientific, USA) was used to measure the concentration and purity of the DNA. The following primers were used for PCR to target the V3–V4 region of the 16S rRNA gene: 5′-ACTCCTACGGGAGGCAGCA-3′ for the forward primer and 5′-GGACTACHVGGGTWTCTAAT-3′ for the reverse primer. The construction of the libraries was performed in two steps. In the first step, DNA was used as a template, and adapter-linked primers were created for PCR amplification. In the second step, the first-step PCR product was used as a template for further PCR amplification. The purpose of incorporating adapter-linked primers was to facilitate the addition of barcodes/indices during the second library preparation step. The amplified products were purified using OMEGA DNA purification columns, and the purified products were subsequently quantified by fluorescence. On an Illumina NovaSeq 6000 platform, sequencing was carried out using the PE250 sequencing method.

After gradient dilution and mixing of each qualifying onboard sequencing library in accordance with the necessary ratio, onboard sequencing was carried out. Illumina NovaSeq was used for paired-end amplicon sequencing.

A PCR negative control was established, with sterile water as the template for amplification. The PCR products of the negative control were detected, and no bands appeared; only then could the subsequent experiments proceed. Bacterial contaminant removal was performed using four paraffin-only samples (no tissue) and reference to the literature. The resulting contaminant-free 16S rRNA profiles were subsampled to 2,000 sequences per sample for downstream comparative analysis.

### Sequencing data processing

To combine the readings from each sample, FLASH v.1.2.11 software (18) was used, with a minimum overlap length of 10 base pairs and a maximum permitted mismatch rate of 0.2 inside the overlap zone. The raw tag data consisted of merged sequences that were obtained. The assembled sequences were subjected to quality filtering using Trimmomatic (version 0.33) (19) and chimera removal using UCHIME (version 8.1) (20) after receiving the raw sequencing data in FASTQ format. This produced high-quality tag sequences.

### OTU/ASV analysis

For operational taxonomic unit (OTU) clustering, sequence clustering was performed using USEARCH (21) (version 10.0) at a 97% similarity level (default setting). OTUs were filtered using a threshold of 0.005% of the total sequenced reads (22).

For amplicon sequence variant (ASV) analysis, for denoising of quality-controlled data, the DADA2 (23) method in QIIME2 (24) (version 2020.6) was utilized. ASVs were filtered at a threshold of 0.005% of the total sequenced reads.

### Microbial analysis

**Fig 1:**
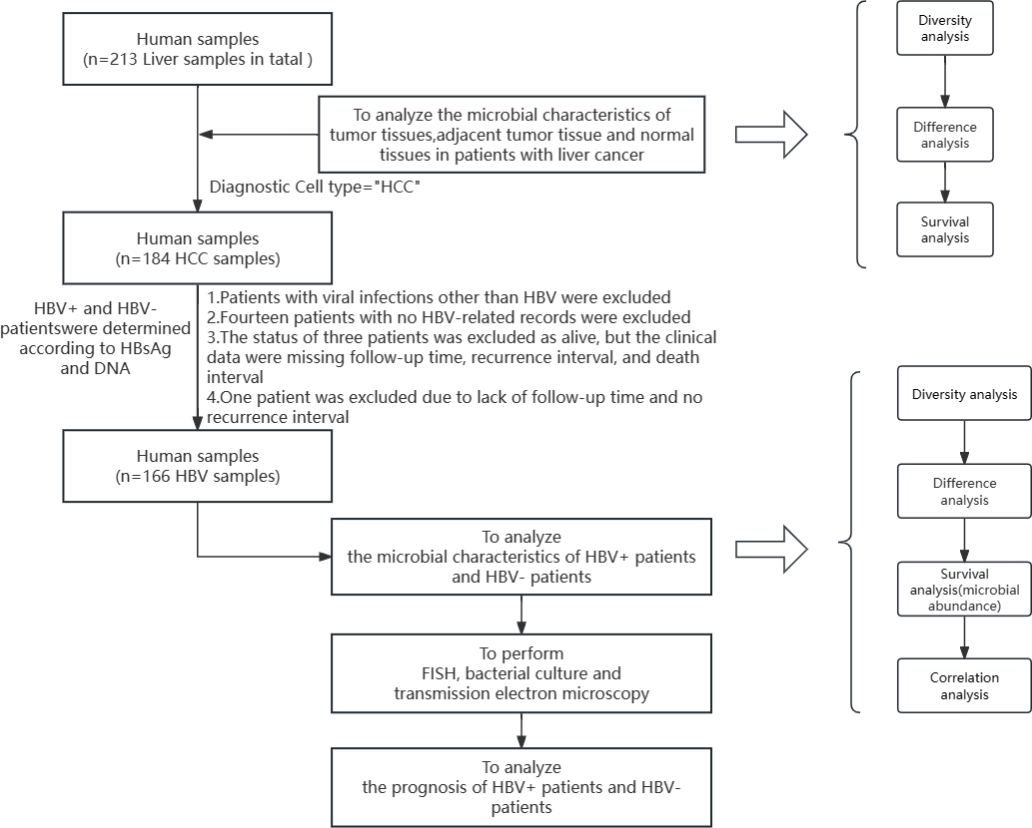
Overall flow chart.

Before the downstream analysis took into account the diversity and composition of microbes, the purified OTU table was resampled to the minimum number of sequences per sample. The R package “vegan” is utilized to determine alpha diversity, which can be expressed as the Shannon index and the Invsimpson index. Nonmetric multidimensional scaling (NMDS) analysis based on weighted UniFrac distance and unweighted UniFrac distance and principal coordinate analysis (PCoA) based on Bray-Curtis distance were used to analyze the diversity. To examine microbiological differences across groups, we used the R package “EdgeR” (25) and the linear discriminant analysis effect size (LEfSe) algorithm (26) (http://huttenhower.sph.harvard.edu/lefse/) for visualization. Based on the Kyoto Encyclopedia of Genes and Genomes and Greengenes databases, PICRUSt (27) was utilized to predict the functional characteristics of microbiomes. STAMP software was used to display differential paths. Kaplan-Meier survival curves and Cox regression were used to investigate the effects of HBV on relapse-free survival (RFS) and overall survival (OS). Both univariate and multivariate regression analyses included traditional prognostic factors for HBV and HCC. A *P*-value <0.05 was considered to indicate statistical significance. False discovery rate (FDR) < 0.05 was considered significant. The R software package “randomForest” (28) was used to construct a random forest model to achieve group prediction and visualize the importance of features. Correlation analysis was performed using the R package “Hmisc” (29), and the microbial network was visualized using the R package “igraph” (30). The overall analysis process is shown in Fig. 1.

First, the microbial characteristics of 213 patients with primary liver cancer were analyzed via diversity analysis (abundance difference analysis of tumor tissue, adjacent tumor tissue, and normal tissue), difference analysis (significant difference analysis between three microorganisms), and survival analysis (microbial prognostic effect analysis). Then, 166 patients with HCC who had no other viral infection other than HBV were further screened, and fluorescence *in situ* hybridization (FISH), bacterial culture, and transmission electron microscopy experiments were performed on the tissues of these patients to determine the bacteria present in the tissues. Then, the microbial characteristics of patients with HCC who were HBV+ or HBV− were analyzed. These analyses included diversity analysis (abundance difference analysis of tumor tissue, adjacent tumor tissue, and normal tissue), difference analysis (significant difference analysis of three microorganisms), survival analysis (impact analysis of microbial prognosis), correlation analysis (microbial interaction analysis), and prognosis analysis of HBV+ and HBV− patients in HCC patients.

### FISH and immunofluorescence

The tissue was dehydrated by gradient alcohol, paraffin, and embedding. The paraffin was cut into slices using a slicer, a slicing machine, and an oven set at 60°C for 2 hours. The sections were soaked for 15 minutes each in two changes of dewaxing transparent liquid. The cells were dehydrated for 5 minutes each in two changes of 100% ethanol. Then, for 5 minutes each, the samples were dehydrated in an ethanol gradient of 85% and 75% ethanol, followed by rinse with diethyl pyrocarbonate-treated water. The slices were incubated in retrieval solution for 10 to 15 minutes (199°F), depending on the duration of tissue fixation, and then allowed to cool naturally. Using a liquid blocker pen, the target tissue was marked in accordance with its properties. The samples were incubated with proteinase K (20 µg/mL) working solution for 15 minutes at 37°C, followed by washing in clean water for 5 minutes, and then three washes in a rocker device with phosphate buffered saline (PBS). After adding 3% methanol-H_2_O_2_, the mixture was incubated at room temperature for 15 minutes in the dark. The slides were gently agitated in PBS three times for 5 minutes each. Prehybridization solution was added to each sample, which was then incubated at 37°C for 1 hour. After removing the prehybridization solution, 1 µM 16S probe hybridization solution was added, and the sections were incubated in a humidity chamber and hybridized at 40°C overnight. The hybridization solution was removed. The sections were washed once in 1× ‌saline-sodium citrate (SSC) buffer for 5 minutes at 37°C, twice in 2× SSC for 10 minutes at 37°C, and once in 0.5× SSC for 10 minutes at room temperature. If there are more hybrids that are not specified, formamide washing can be added. After the blocking solution was added to the area, the samples were allowed to sit at room temperature for half an hour. After removing the blocking solution, mouse anti-digoxigenin-labeled peroxidase was added. After 40 minutes, the cells were incubated at 37°C and then washed with PBS four times for 5 minutes each. After the sections were slightly dried, the newly made tyramide chromogenic reagent was added to the marked tissue. The mixture was allowed to react for 5 minutes at room temperature in the dark. The pieces were then washed with PBS three times for 10 minutes each. The remaining probes and chromogenic reagents were added. HBV is paired with Cy3-tyramide, while nonspecific pairing with iF647-tyramide has been employed. After 8 minutes of incubation in the dark with 4′,6-diamidino-2-phenylindole (DAPI), the samples were mounted. A positive fluorescence microscope was used to capture images. When UV light is applied at wavelengths of 330 nm–380 nm and 420 nm, DAPI fluoresces blue; when light is applied at wavelengths of 465 nm–495 nm and 515 nm–555 nm, 5-carboxyfluorescein fluoresces green; and when light is applied at wavelengths of 510 nm–560 nm and 590 nm, CY3 fluoresces red.

The technique of immunofluorescence was used. To enable antibodies to bind to the appropriate epitopes, the slides were first cleaned with 0.1 M Tris-HCl and then permeabilized with 0.3% Triton X. The slides were subsequently blocked with 2% fetal bovine serum for 30 minutes at room temperature to achieve the best specificity and sensitivity. The slides were then subjected to incubation with primary antibodies (anti-lipopolysaccharide and anti-lipoteichoic acid) overnight at 4°C, followed by incubation with fluorophore-conjugated secondary antibodies for 1 hour at room temperature. Nucleic acid counterstaining was accomplished using DAPI at room temperature. A fluorescence microscope (Nikon A1, Tokyo, Japan) was used to take pictures of the stained slides after they had been mounted in DAPI antifade solution in a dark room and cleaned.

Probe information was 16S EUB338 (5′-GCTGCCTCCCGTAGGAGT-3′), HBV (5‘-CAACAGGAGGGATACATAGAGGTTCCTTGAGCAGT-3′), and nonspecific (5′-CGACGGAGGGCATCCTCA-3′).

## RESULTS

### Demographic characteristics of the participants

Table 1 presents the clinical details of the patients included in the study. Samples were collected from 213 patients who had undergone inclusion and exclusion procedures. The median age of the patients was 54.9 years, with an age range of 23 to 79 years. Of the 213 patients, 177 (83.1%) were male. In terms of diagnosis, 184 patients (86.4%) were identified with the HCC cell type. Nine patients receive chemotherapy as part of their treatment regimen. Additionally, 93 patients (47.4%) were found to be without HBV infections, while 88.6% tested positive for hepatitis B surface antigen (HBsAg), and 11.4% tested negative. Vascular invasion was observed in 29.3% of the patients, while the remaining 70.7% showed no evidence of vascular invasion. Lymph node invasion was present in 7.11% of patients, and only 92.9% showed no lymph node invasion. Tumor differentiation varied among patients, with 25.4% exhibiting high differentiation and 74.6% showing low differentiation. The majority of patients (91.2%) had single tumors, whereas 8.81% had multiple tumors. The tumor size was greater than or equal to 5 cm in 53.5% of patients and less than 5 cm in 46.5% of patients.

**TABLE 1:** Baseline characteristics of patients with primary liver cancer^*a*^

Characteristic	Value
Age (*n* = 213)	54.9 (23–79)
Gender (*n* = 213)	
Female	36 (16.9%)
Male	177 (83.1%)
Status (*n* = 213)	
Alive	119 (55.9%)
Dead	94 (44.1%)
Chemotherapy (*n* = 213)	
No	204 (95.8%)
Yes	9 (4.23%)
Diagnostic cell type (*n* = 213)	
ICC	14 (6.57%)
ICC, Other	1 (0.47%)
HCC	184 (86.4%)
HCC, mixed type	1 (0.47%)
Mixed type	9 (4.23%)
Other	4 (1.88%)
Vascular invasion (*n* = 208)	
Abnormal	61 (29.3%)
Normal	147 (70.7%)
Tumor differentiation (*n* = 213)	
High	54 (25.4%)
Low	159 (74.6%)
Tumor number (*n* = 159)	
Single	145 (91.2%)
Multiple	14 (8.81%)
Tumor size (*n* = 159)	
≥5 cm	85 (53.5%)
<5 cm	74 (46.5%)
Lymph node invasion (*n* = 211)	
No	196 (92.9%)
Yes	15 (7.11%)
HBsAg (*n* = 210)	
Abnormal	186 (88.6%)
Normal	24 (11.4%)
CA125 (*n* = 188)	
Abnormal	76 (40.4%)
Normal	112 (59.6%)
ALT (*n* = 173)	
Abnormal	22 (12.7%)
Normal	151 (87.3%)
AST (*n* = 209)	
Abnormal	95 (45.5%)
Normal	114 (54.5%)
GGT (*n* = 209)	
Abnormal	83 (39.7%)
Normal	126 (60.3%)
AFP (*n* = 192)	
Abnormal	187 (97.4%)
Normal	5 (2.60%)
CEA (*n* = 200)	
Abnormal	14 (7.00%)
Normal	186 (93.0%)
Hb (*n* = 208)	
Abnormal	47 (22.6%)
Normal	161 (77.4%)
PDW (*n* = 206)	
Abnormal	108 (52.4%)
Normal	98 (47.6%)
PT (*n* = 211)	
Abnormal	28 (13.3%)
Normal	183 (86.7%)
INR (*n* = 211)	
Abnormal	12 (5.69%)
Normal	199 (94.3%)
HBV (*n* = 196)	
HBV−	93 (47.4%)
HBV+	103 (52.6%)

^*a*^ AFP, alpha-fetoprotein; PDW, platelet distribution width; ALT, alanine aminotransferase; AST, aspartate aminotransferase; GGT, γ-glutamyl transpeptidase; CEA, carcinoembryonic antigen; hb, hemoglobin; PT, Prothrombin Time; INR, international normalized ratio.

### Different microbiota profiles in tumor tissue, adjacent tumor tissue, and normal tissue

To investigate tissue-specific differences in microbial composition, we conducted pairwise comparisons of microbial diversity between tumor tissue, adjacent tumor tissue, and normal tissue from the same cohort of 213 patients with liver cancer. Each pairwise comparison (tumor vs normal; tumor vs adjacent tumor; adjacent tumor vs normal) was independently assessed using the same diversity metrics and statistical thresholds. In addition, a three-group comparison was performed to provide an overall view of microbial heterogeneity across tissue types. This stepwise comparison strategy enabled us to identify both global and tissue-specific patterns in alpha and beta diversity while ensuring consistency in sequencing depth, processing, and analytical methods.

Alpha diversity was evaluated using multiple metrics, including PD_whole_tree, Shannon index, and Invsimpson index, each capturing different aspects of microbial richness or evenness. Beta diversity was assessed using weighted and unweighted UniFrac distances, visualized with both PCoA and NMDS. Although some comparisons did not reach statistical significance, we report all results for transparency, regardless of significance, to avoid selective reporting.

**Fig 2:**
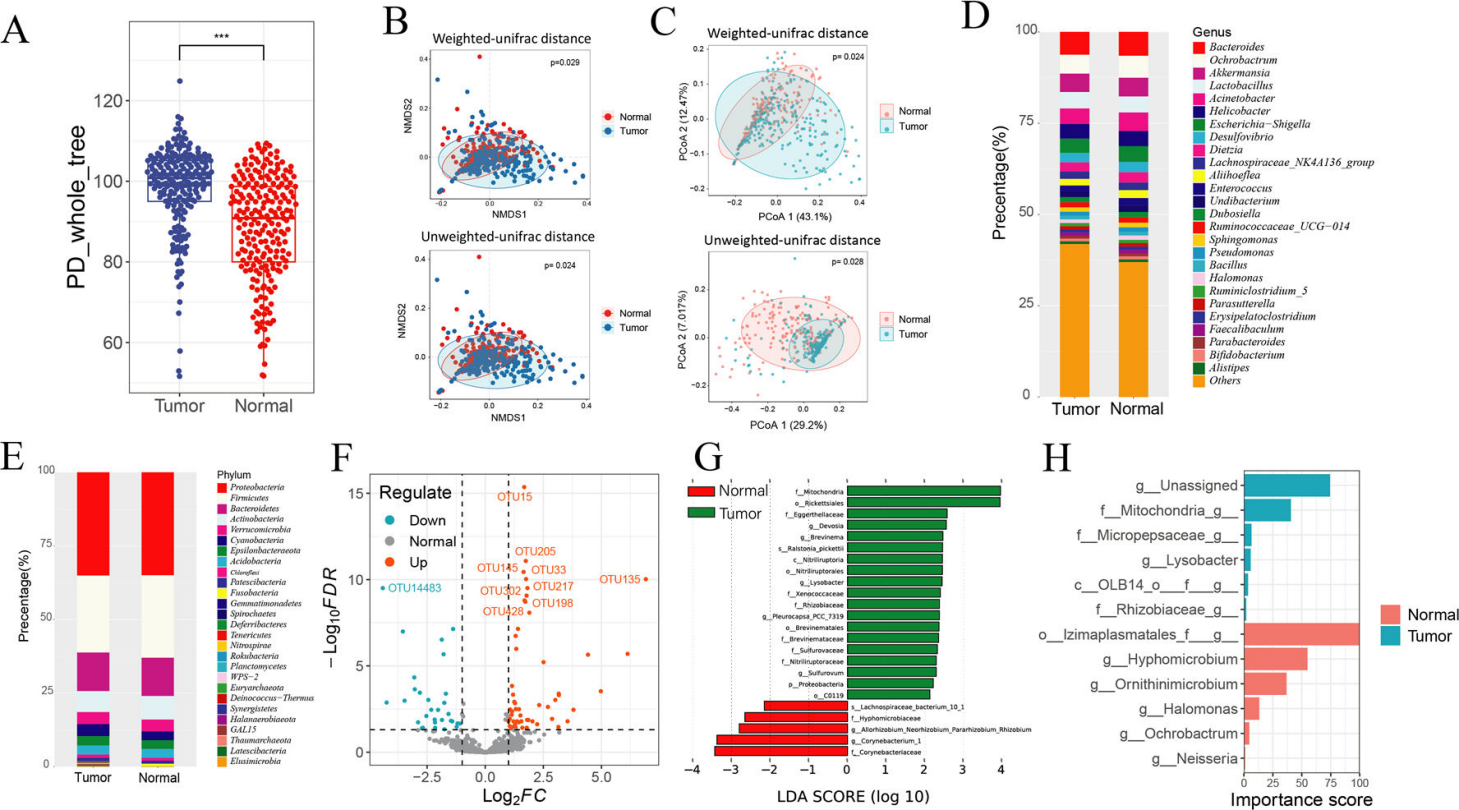
Characteristics of the microbiomes of tumor tissue and normal tissue from patients with primary liver cancer. (**A**) Box plots of the PD_whole_tree index of tumor tissue and normal tissue. (**B**) NMDS based on weighted and unweighted UniFrac distances between tumor and normal tissues. (**C**) PCoA of tumor and normal tissues. (**D**) Stacked bar charts of the 27 taxa with the highest relative abundance in tumor tissue and normal tissue at the phylum level. (**E**) Stacked histogram of the 27 taxa with the highest relative abundances in tumor tissue and normal tissue at the genus level. (**F**) Volcano plot of OTU abundance differences between tumor and normal tissues. The cutoff was set at |log2 fold change| > 1 and *P* < 0.05. OTUs significantly enriched in tumor tissue are shown in red, while those significantly reduced in tumor tissue (i.e., enriched in normal tissue) are shown in green. (**G**) LEfSe analysis identifying bacterial taxa that differ significantly in abundance between tumor and normal tissues. Only taxa with linear discriminant analysis (LDA) score (log10) > 2.0 and *P* < 0.05 were considered significant. Taxa enriched in tumor tissue are shown in green, and those enriched in normal tissue are shown in red.(**H**) Bar plot of the top 20 genera contributing to the discrimination between tumor and normal tissues based on the random forest model. Bar colors represent the group in which each genus is more relatively abundant: cyan indicates enrichment in tumor tissue, and pink indicates enrichment in normal tissue.

Tumor samples, adjacent tumor samples, and normal samples from 213 patients with liver cancer were studied. The comparison of gut microbial diversity in different tissues was assessed using alpha and beta diversity measures. Differences were observed in the alpha diversity metric PD_whole_tree (Fig. 2A), whereas other alpha diversity metrics, including the Invsimpson index and Shannon index, among others, exhibited no significant differences (Fig. S1A). Beta diversity calculations, using weighted UniFrac distance and unweighted UniFrac distance, also revealed no significant differences between these groups (Fig. S1B, *P* = 0.069; *P* = 0.075). Additionally, PCoA did not reveal significant differences between tumor tissue, adjacent tumor tissue, or normal tissue (Fig. S1C, *P* = 0.072; *P* = 0.067).

In a separate analysis, the tumor tissue and normal tissue from 213 patients with primary liver cancer were compared. There were no significant differences in the alpha diversity between tumor and normal tissues (Fig. 2A). However, beta diversity calculations, using the weighted UniFrac distance and unweighted UniFrac distance, revealed significant differences between tumor and normal tissues (Fig. 2B, *P* = 0.029; *P* = 0.024). PCoA also revealed significant differences between these two groups (Fig. 2C, *P* = 0.024; *P* = 0.028).

Further analysis of tumor tissue and adjacent tumor tissue from 213 patients with primary liver cancer was conducted. The alpha diversity indices, Shannon index and Invsimpson index, showed no significant differences between the tumor and adjacent tumor tissues (Fig. S1D, *P* = 0.57; *P* = 0.47). Beta diversity calculations using the weighted UniFrac distance and unweighted UniFrac distance revealed significant differences between tumors and adjacent tumor tissues (Fig. S1E, *P* = 0.034; *P* = 0.03). PCoA also revealed significant differences between these two groups (Fig. S1F, *P* = 0.042; *P* = 0.048).

Finally, adjacent tumor tissue and normal tissue from 213 patients with primary liver cancer were compared. The alpha diversity indices, Shannon index and Invsimpson index, showed no significant differences between adjacent tumor tissue and normal tissue (Fig. S1G, *P* = 0.89; *P* = 0.86). Beta diversity calculations using the weighted UniFrac distance and unweighted UniFrac distance also revealed no significant differences between these groups (Fig. S1H, *P* = 0.983; *P* = 0.983). PCoA did not reveal significant differences between the two groups (Fig. S1I, *P* = 0.985; *P* = 0.986).

A stacked bar chart was created for the relative abundances of the top 27 bacteria at the phylum, class, order, family, genus, and species levels (Fig. S1J). In general, eight bacterial phyla (*Proteobacteria*, *Firmicutes*, *Bacteroidetes*, *Actinobacteria*, *Verrucomicrobia*, *Cyanobacteria*, *Epsilonbacteraeota*, and *Acidobacteria*) accounted for up to 90% of the bacteria in the top 27 relative abundances and were dominant in tumors, adjacent tumor tissues, and normal tissues (Fig. 2D). Moreover, four genera (*Bacteroides*, *Ochrobactrum*, *Akkermansia*, and *Lactobacillus*) accounted for approximately 20% of the top 27 bacteria in terms of relative abundance and predominated in tumors, adjacent tumor tissues, and normal tissues (Fig. 2E). At the genus level, four genera—*Bacteroides*, *Ochrobactrum*, *Akkermansia*, and *Lactobacillus*—accounted for approximately 20% of the cumulative abundance within the top 27 genera identified across tumor, adjacent tumor, and normal tissues (Fig. 2D). Several genera showed statistically significant differences in relative abundance between HBV+ and HBV− patients; however, most of these differentially abundant genera exhibited moderate overall abundance, collectively accounting for approximately 5% of the total microbial community. These results indicate that while the observed differences are statistically significant, their biological impact may be relatively limited compared to the dominant taxa. At the phylum level, eight phyla—*Proteobacteria*, *Firmicutes*, *Bacteroidetes*, *Actinobacteria*, *Verrucomicrobia*, *Cyanobacteria*, *Epsilonbacteraeota*, and *Acidobacteria*—collectively comprised approximately 90% of the bacterial community across all tissue types (Fig. 2E), indicating that the microbial composition is dominated by a small number of highly abundant phyla.

EdgeR was used to analyze the difference between tumor tissue and normal tissue to identify OTUs with significant differential expression. Analysis revealed that compared with those in normal tissues, three OTUs were enriched in tumor tissues, 25 OTUs were decreased, and a total of 28 OTUs, including those of the phyla *Latescibacteria* and genera such as *Sphingobium* and *Erythrobacter*, were significantly expressed (Fig. 2F). Compared with those in normal tissues, three OTUs were enriched in adjacent tumor tissues, and two OTUs were decreased; that is, five OTUs, including genera such as *Asaia* and *Acidisphaera*, were significantly differentially expressed (Fig. S1K). Compared with those in adjacent tumor tissues, 20 OTUs were enriched in tumor tissues, and one OTU decreased; that is, 21 OTUs, including genera such as *Sphingobium* and *Hydrogenophaga*, were significantly differentially expressed (Fig. S1L).

LEfSe analysis was performed to identify differentially abundant bacterial taxa across tissue types. To provide a comprehensive view of microbial shifts, taxa at both the phylum and genus levels were examined. For instance, *Proteobacteria* (phylum level) was enriched in tumor tissues, while genera such as *Devosia*, *Brevinema*, *Lysobacter*, *Pleurocapsa*_PCC-7319, *Sulfurovum*, *Allorhizobium-Neorhizobium-Pararhizobium-Rhizobium*, and *Corynebacterium*_1 were also identified as significantly different between tumor and normal tissues (Fig. 2G). The selection of different taxonomic levels was intended to capture both broad (phylum-level) and more specific (genus-level) microbial differences associated with tissue type. *Acidothermus*, *Gilliamella*, *Mycoplasma*, *Lautropia*, CL500-29_marine_group, *Ruminococcaceae*_UCG-005, *Gemella*, *Rothia*, and *Corynebacterium*_1 contributed to the differences between tumors and adjacent tumor tissues (Fig. S1M). The genera *Exiguobacterium*, *Gemella*, *Megasphaera*, *Kocuria*, *Pseudoalteromonas*, *Truepera*, *Hafnia-Obesumbacterium*, *Pleurocapsa*_PCC-7319, JGI_0001001-H03, *Nitrolancea*, *Rodentibacter*, and the OM43_clade are involved in interpreting the differences between adjacent tumor tissue and normal tissue (Fig. S1N).

In addition, we used genera of bacteria to establish random forest classifiers and conducted fivefold cross-validation to distinguish tumor tissue from normal tissue. The data set was divided into training and test sets at a ratio of 7:3. The bar chart displays the top 20 most important features in the genus groups for distinguishing tumor and normal tissues (Fig. 2H). The model, established by genus flora, achieved an area under the curve (AUC) of 1 in the training set and 0.556 in the test set (Fig. S1O and P). The general accuracy of the three generic microbial features in distinguishing tumors from normal tissue was 52.21% (Fig. S1Q).

### HBV of the intratumoral microbiome

Subsequently, we further analyzed HCC patients who had no viral infections other than HBV. In this study, 166 participants were divided into two groups: 72 (44%) with HBV DNA levels below 2,000 IU/mL, labeled as HBV−, and 94 (56%) with HBV DNA levels at or above 2,000 IU/mL, labeled as HBV+. From the initial group, we excluded 47 patients: 14 due to insufficient HBV DNA data, 29 because they did not have pure HCC samples, and four due to incomplete survival data. Table 2 presents the clinical information for the remaining patients. The sample comprised 166 patients who had undergone the inclusion/exclusion processes. The median age was 54.36 years (range 23–79), with 140 (84.3%) being male. Five patients did not receive chemotherapy. HBsAg levels were positive in 91.6% and negative in 8.43% of patients. Vascular invasion was abnormal in 27.4% and normal in 72.6% of patients. Lymph node invasion was abnormal in 97.6% of patients and normal in 2.44%. Tumor differentiation was high in 22.3% and low in 77.7% of patients. The tumor multiplicity was single in 89.9% and multiple in 10.1%. The tumor size was 5 cm or larger in 55.0% of patients and smaller than 5 cm in 45.0%.

**TABLE 2:** Baseline characteristics of HCC patients with HBV infection^*a*^

Characteristic	Value
Age (*n* = 166)	54.36 (23–79)
Gender (*n* = 166)	
Female	26 (15.7%)
Male	140 (84.3%)
Status (*n* = 166)	
Alive	91 (54.8%)
Dead	75 (45.2%)
Chemotherapy (*n* = 166)	
No	161 (97.0%)
Yes	5 (3.01%)
Vascular invasion (*n* = 164)	
Abnormal	45 (27.4%)
Normal	119 (72.6%)
Tumor differentiation (*n* = 166)	
High	37 (22.3%)
Low	129 (77.7%)
Tumor number (*n* = 129)	
Single	116 (89.9%)
Multiple	13 (10.1%)
Tumor size (*n* = 129)	
≥5 cm	71 (55.0%)
<5 cm	58 (45.0%)
Lymph node invasion (*n* = 164)	
No	160 (97.6%)
Yes	4 (2.44%)
HBsAg (*n* = 166)	
Positive	152 (91.6%)
Negative	14 (8.43%)
CA125 (*n* = 148)	
Abnormal	55 (37.2%)
Normal	93 (62.8%)
ALT (*n* = 165)	
Abnormal	47 (28.5%)
Normal	118 (71.5%)
AST (*n* = 165)	
Abnormal	72 (43.6%)
Normal	93 (56.4%)
AFP (*n* = 165)	
Abnormal	38 (23.0%)
Normal	127 (77.0%)
CEA (*n* = 158)	
Abnormal	9 (5.70%)
Normal	149 (94.3%)
Hb (*n* = 164)	
Abnormal	36 (22.0%)
Normal	128 (78.0%)
PDW (*n* = 162)	
Abnormal	84 (51.9%)
Normal	78 (48.1%)
PT (*n* = 166)	
Abnormal	24 (14.5%)
Normal	142 (85.5%)
INR (*n* = 166)	
Abnormal	9 (5.42%)
Normal	157 (94.6%)
HBV (*n* = 166)	
HBV−	72 (43.4%)
HBV+	94 (56.6%)

^*a*^ AFP, alpha-fetoprotein; PDW, platelet distribution width.

Table 3 shows the relationships between HBV+ and HBV− and clinical factors. The results showed that HBV+ patients had a tumor size ≥5 cm, HBsAg was positive, CA125, GGT, and alpha-fetoprotein (AFP) were normal, and ALT, AST, Hb, platelet distribution width (PDW), PT, and INR were abnormal (*P* < 0.05).

**TABLE 3:** Baseline characteristics of patients classified as HBV+ and HBV−^*a*^

Characteristic	Value for HBV− patients (*N* = 72)	Value for HBV+ patients (*N* = 94)	*P*
Age	55.8 (10.8)	53.3 (12.1)	0.161
Gender			0.444
Female	9 (12.5%)	17 (18.1%)	
Male	63 (87.5%)	77 (81.9%)	
Status			0.113
Alive	45 (62.5%)	46 (48.9%)	
Dead	27 (37.5%)	48 (51.1%)	
Chemotherapy			1
No	70 (97.2%)	91 (96.8%)	
Yes	2 (2.78%)	3 (3.19%)	
Vascular invasion			0.187
Abnormal	24 (33.3%)	21 (22.8%)	
Normal	48 (66.7%)	71 (77.2%)	
Tumor differentiation			0.56
High	14 (19.4%)	23 (24.5%)	
Low	58 (80.6%)	71 (75.5%)	
Tumor number			0.108
Single	59 (95.2%)	57 (85.1%)	
Multiple	3 (4.84%)	10 (14.9%)	
Tumor size			**0.007**
≥5 cm	26 (41.9%)	45 (67.2%)	
<5 cm	36 (58.1%)	22 (32.8%)	
Lymph node invasion			0.632
No	71 (98.6%)	89 (96.7%)	
Yes	1 (1.39%)	3 (3.26%)	
HBsAg			**<0.001**
Positive	58 (80.6%)	94 (100%)	
Negative	14 (19.4%)	0 (0.00%)	
CA125			**0.006**
Abnormal	33 (50.0%)	22 (26.8%)	
Normal	33 (50.0%)	60 (73.2%)	
ALT			**0.015**
Abnormal	13 (18.1%)	34 (36.6%)	
Normal	59 (81.9%)	59 (63.4%)	
AST			**0.005**
Abnormal	22 (30.6%)	50 (53.8%)	
Normal	50 (69.4%)	43 (46.2%)	
GGT			**0.008**
Abnormal	9 (12.5%)	29 (31.2%)	
Normal	63 (87.5%)	64 (68.8%)	
AFP			1
Abnormal	64 (98.5%)	89 (98.9%)	
Normal	1 (1.54%)	1 (1.11%)	
CEA			0.505
Abnormal	5 (7.25%)	4 (4.49%)	
Normal	64 (92.8%)	85 (95.5%)	
Hb			**0.017**
Abnormal	9 (12.5%)	27 (29.3%)	
Normal	63 (87.5%)	65 (70.7%)	
PDW			**0.015**
Abnormal	45 (63.4%)	39 (42.9%)	
Normal	26 (36.6%)	52 (57.1%)	
PT			**0.029**
Abnormal	5 (6.94%)	19 (20.2%)	
Normal	67 (93.1%)	75 (79.8%)	
INR			**0.005**
Abnormal	0 (0.00%)	9 (9.57%)	
Normal	72 (100%)	85 (90.4%)	

^*a*^ Statistically significant *P*-values are in bold (*P *< 0.05).

**Fig 3:**
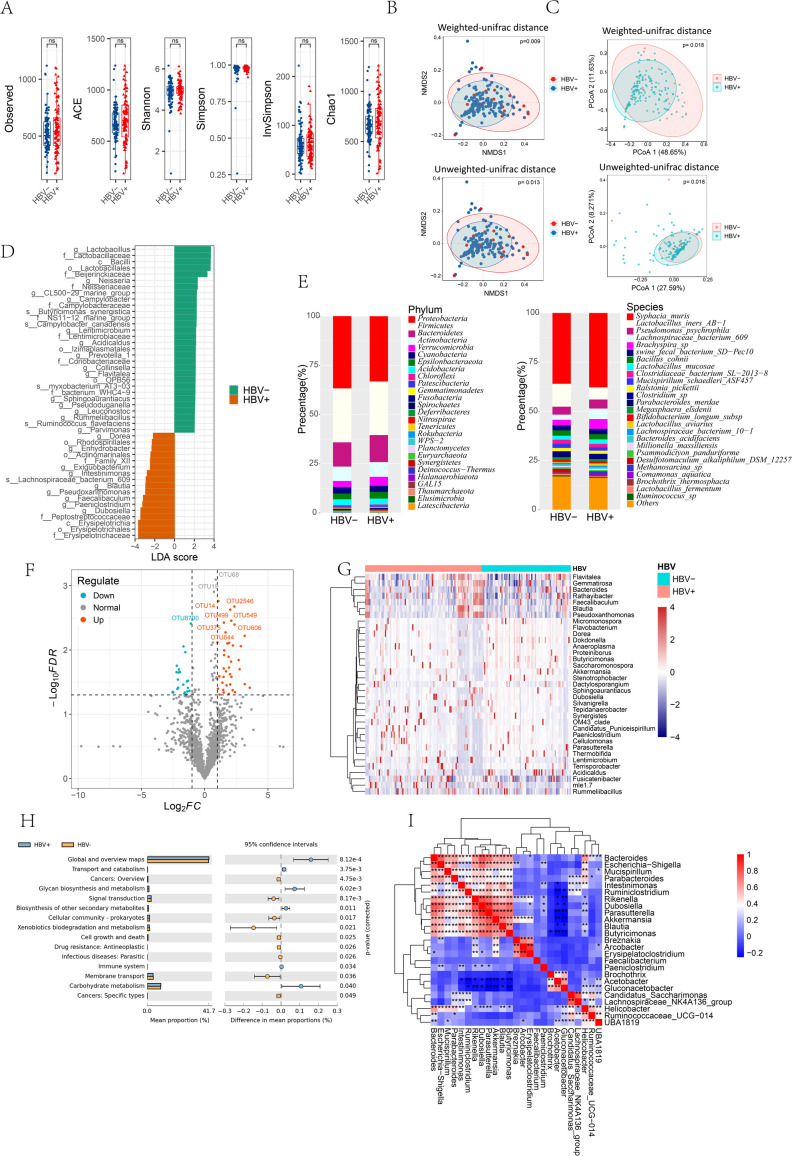
Microbiological characteristics of tumor tissues from patients with HBV+ and HBV− HCC. (**A**) Box plots of the Shannon index and Invsimpson index in tumor tissue from HBV+ and HBV− patients. (**B**) The tumor tissue of HBV+ and HBV− patients was used to calculate the weighted and unweighted UniFrac distances for the NMDS. (**C**) PCoA of tumor samples from HBV+ and HBV-positive patients. (**D**) A histogram of taxa with varying abundances obtained from LEfSe analysis for HBV+ and HBV-positive patients. *P* < 0.05 and linear discriminant analysis (LDA) score (log10) >2.0 were the only features. (**E**) Stacked bar charts showing the relative abundance at the phylum, class, order, family, genus, and species levels of the 27 taxa found in tumor tissue from HBV+ and HBV-positive patients. (**F**) Volcano plot of the abundance of generic microorganisms in the tumor tissues of patients who were HBV+ or HBV−. The truncation condition was (|log2 fold change| > 1, *P* < 0.05), and the bacteria significantly reduced in the tissue are shown in green. Bacteria that were significantly enriched in the tissue are shown in red. (**G**) Heatmap analysis was performed for the relative abundance of HBV+ and HBV− microbiome differences at the genus level, and the Z score and *P*-value were calculated and are displayed in the heatmap (** represents *P* < 0.01 and * represents 0.01 < *P* < .05). (**H**) Bar chart illustrating the pathways associated with the differential functions of HBV+ and HBV− in HCC patients. Welch’s *t*-test was used for comparisons, and only paths with a Benjamini-Hochberg false discovery rate of less than 0.05 are displayed. (**I**) Heatmaps showing Pearson correlation coefficients between selected bacterial genera in HBV− and HBV+ patients, based on genus-level relative abundance data. Colors indicate the strength and direction of pairwise correlations (red: positive; blue: negative). The heatmaps highlight distinct correlation patterns in microbial communities between HBV− and HBV+ individuals.

The alpha and beta diversity of the microbiota of HBV+ and HBV− patients were compared. The alpha diversity, as measured by the Shannon index and the anti-Simpson index, was not significantly different between the HBV+ and HBV− patients (Fig. 3A, *P* = 0.068, *P* = 0.59). Beta diversity calculations, using weighted and unweighted UniFrac distances, revealed significant differences between HBV+ and HBV− patients (Fig. 3B, *P* = 0.009; *P* = 0.013). Additionally, PCoA demonstrated significant differences between the two groups (Fig. 3C, *P* = 0.018; *P* = 0.018). The relative abundances of the top 27 bacteria at the phylum, class, order, family, genus, and species levels were plotted in stacked bar charts (Fig. 3D). Four genera (*Bacteroides*, *Ochrobactrum*, *Akkermansia*, and *Lactobacillus*) comprised approximately 20% of the top 27 bacteria in terms of relative abundance and were prevalent in both HBV+ and HBV− patients. Fourteen species of bacteria (*Syphacia muris*, *Lactobacillus iners* AB-1, *Pseudomonas psychrophila*, *Lachnospiraceae bacterium* 609, *Brachyspira* sp., swine fecal bacterium sd-pec10, *Bacillus cohnii*, *Lactobacillus mucosae*, *Clostridiaceae bacterium* SL-2013-8, *Mucispirillum schaedleri* ASF457, *Ralstonia pickettii*, *Clostridium* sp., *ParaBacteroides merdae*, and *Megasphaera elsdenii*) accounted for approximately 75% of the top 27 most abundant bacteria and were dominant in both HBV− and HBV+ patients.

EdgeR was used to analyze the differences between HBV+ and HBV− patients to identify bacteria at the phylum, class, order, family, genus, and species levels with significantly different expression. Compared with HBV− patients, HBV+ patients were enriched in 3 families, 31 genera, and 12 species of bacteria, while they were depleted in 4 families, 4 genera, and 9 species. There were 7 families (Fig. S2A), 35 genera (Fig. 3F), and 21 species (Fig. S2A) of bacteria whose expression significantly differed. A heatmap (Fig. 3G), generated by clustering based on the abundance of significantly expressed genera, clearly depicted the microbiome differences between the HBV+ and HBV− patients.

LEfSe can be used to analyze bacterial signatures between different tissues and identify groups that significantly distinguish HBV+ from HBV−. *Desulfovibrio*, *Dietzia*, *Pseudoxanthomonas*, *Enhydrobacter*, *Eubacterium _nodatum_group*, *Acinetobacter*, *Aliihoeflea*, *Delftia*, *Desulfovibrio*, *Dietzia*, *Pseudoxanthomonas*, *Halomonas*, *Leuconostoc*, *Ochrobactrum*, *Reyranela, Bacteroides*_plebeius, *Catellicoccus_marimammalium*_M35_04_3, *Myxobacterium*_AT3-03, *Lachnospiraceae_bacterium*_609, et al. help explain the difference between HBV+ and HBV− (Fig. 3E).

We used PICRUSt to align the 16S rDNA sequences to the appropriate genes and pathways to examine the functional capacities of the intratumor microbiota in HCC. Figure 3H indicates that the intratumoral microbiome in HCC tissues demonstrated differential enrichment of environmental information processing, metabolism, and cellular functions between the HBV+ and HBV− groups according to PICRUSt taxonomic functional connections. According to the PICRUSt classification function prediction, HBV+ exhibited greater enrichment in pathways associated with glycan biosynthesis and metabolism, global and overview mapping, and carbohydrate metabolism. HBV− exhibited greater enrichment in pathways associated with xenobiotic biodegradation and metabolism, membrane transport, and cancer (Fig. 3H).

In addition, we used generic bacteria to establish random forest classifiers and performed fivefold cross-validation to distinguish between HBV+ and HBV− patients. The data set was divided into a training set and a test set, with a training set-to-test set ratio of 7:3. The bar chart shows the top 20 most important features in the genus group for distinguishing HBV+ and HBV− patients. The overall accuracy of differentiating HBV+ from HBV− patients using 27 generic microbial characteristics was 61.72% (Fig. S2F).

Correlation analysis was conducted to examine co-variation patterns among bacterial genera in HCC patients. Based on relative abundance data, we calculated Pearson correlation coefficients between genera separately in HBV− and HBV+ patients. The results showed that several genera exhibited moderate to strong positive correlations (*R* > 0.5, *P* < 0.05), including *Bacteroides*, *Erysipelatoclostridium*, *Akkermansia*, *Ruminiclostridium*, *Parasutterella*, *Butyricimonas*, *Rikenella*, *Dubosiella*, *Blautia, Intestinimonas*, *Escherichia-Shigella*, *ParaBacteroides*, *Helicobacter*, UBA1819, *Ruminococcaceae_*UCG-014, and *Mucispirillum*. These correlations suggest shared abundance patterns among these genera. A heatmap was used to visualize pairwise correlations, stratified by HBV status (Fig. 3I; Fig. S2H), revealing distinct co-occurrence patterns in HBV− versus HBV+ patients.

### FISH reveals the presence of HBV and intracellular bacteria in tissues

**Fig 4:**
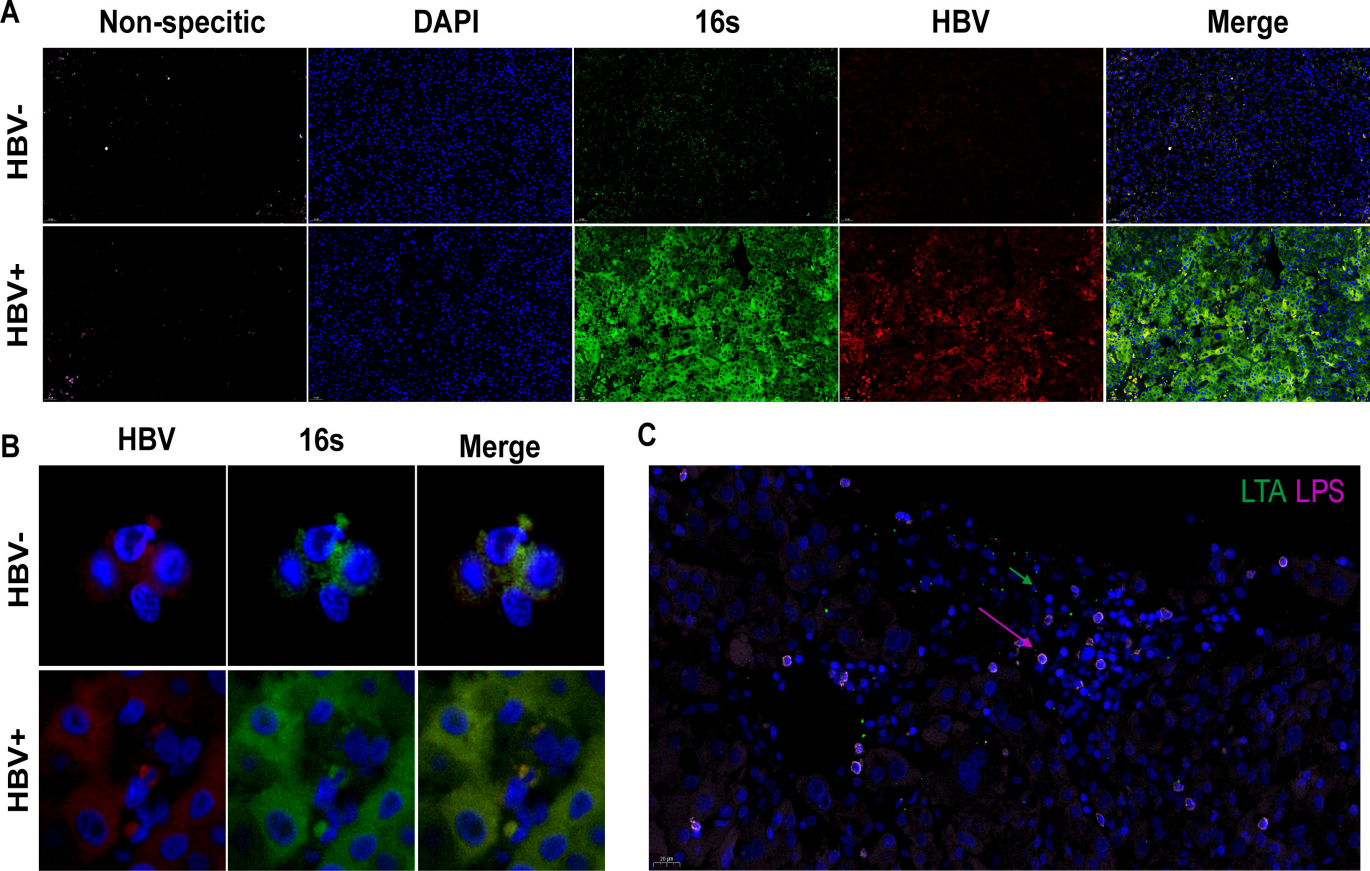
The presence of the microbiota in HBV^High^ and HBV^Low^ HCC patients. (**A**) Staining of bacterial 16S (FAM(488), green), HBV (cy3, red), and nonspecific complement (cy5, pink); the nuclei were counterstained with DAPI. (**B**) Staining of bacterial 16S(FAM(488), green) and HBV (cy3, red); the nuclei were counterstained with DAPI. (**C**) Staining of gram-positive bacteria (FAM(488), green) and gram-negative bacteria (cy5, pink); the nuclei were counterstained with DAPI.

Previously, we utilized 16S rRNA sequencing to identify the presence of bacterial DNA within HCC tissues. Subsequently, we examined the bacterial and HBV DNA content in these tissues using FISH with specific oligonucleotide probes for both bacteria and HBV. We created probes targeting HBV and the 16S rRNA of bacteria and applied them to HCC tissue sections. FISH fluorescence labeling demonstrated the presence of bacterial DNA and HBV DNA in HCC tissues, indicating a connection between HBV infection and increased bacterial levels (Fig. 4A and B). Our imaging also revealed various microorganisms, including both gram-positive and gram-negative microorganisms (Fig. 4C).

### Influence of HBV on HCC prognosis

**Fig 5:**
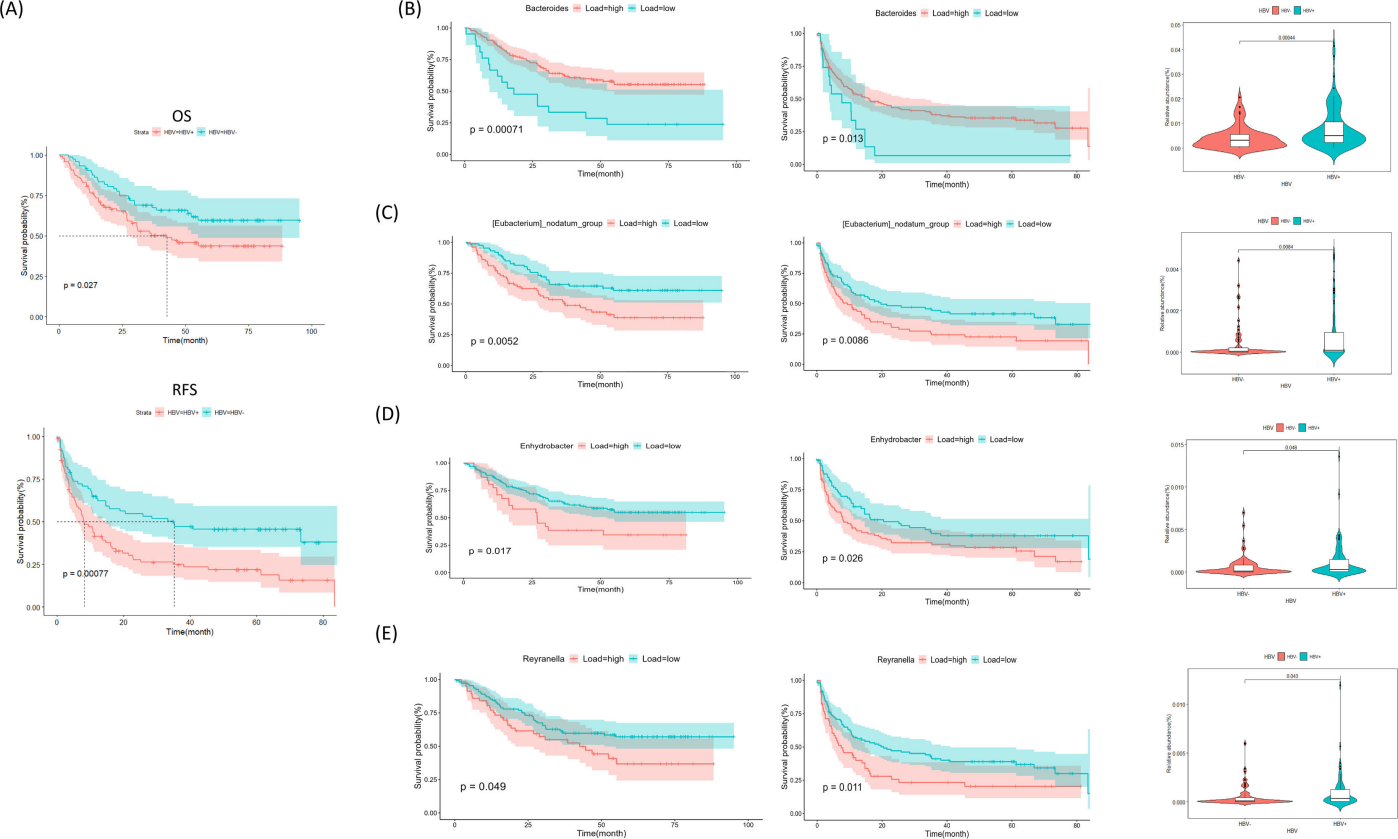
Prognostic analysis of HBV+ and HBV− patients with HCC. (**A**) Kaplan-Meier survival curves for HBV+ and HBV− HCC patients. The OS and RFS were compared using the log-rank test (*P* = 0.027 for OS, *P* = 0.00077 for RFS). (**B**) EdgeR analysis comparing survival curves of bacteria with different relative abundances in tumor tissues of HBV+ and HBV− patients. A *t*-test was used to assess the difference in relative abundance between groups for both OS and RFS. (**C–E**) LEfSe analysis to identify bacteria with different horizontal abundances in tumor tissues of HBV+ and HBV− patients. The left, middle, and right panels represent OS-based survival analysis, RFS-based survival analysis, and relative abundance comparison, respectively. Statistical comparisons were performed using a *t*-test.

We evaluated the survival outcomes of patients with HBV+ and HBV− status. According to Kaplan-Meier analysis, patients who were HBV+ had poorer outcomes (Fig. 5A). The mean OS for HBV+ patients was 34 months (standard deviation: 24 months), while it was 45 months (standard deviation: 24 months) for HBV− patients (*P* = 0.027). RFS showed similar trends, with a mean of 17 months (standard deviation: 22 months) for the HBV+ patients and 32 months (standard deviation: 27 months) for the HBV− patients (*P* = 0.00077). Cox regression analysis demonstrated that HBV status, when considered alongside other significant prognostic factors, such as vascular invasion, sex, chemotherapy, AFP, PDW, lymph node invasion, HBsAg status, tumor size, number, and differentiation, is a predictive marker for OS and RFS (hazard ratio [HR] = 1.95, 95% confidence interval [CI]: 1.31–2.89, *P* = 0.001; Table S1; HR = 1.69, 95% CI: 1.05–2.71, *P* = 0.029; Table 4). Univariate Cox regression analysis supported the predictive significance of HBV status for RFS, with the results approaching statistical significance (HR = 1.6, 95% CI: 0.98–2.61, *P* = 0.06; Table 5).

**TABLE 4:** Univariate Cox regression analysis for the prediction of RFS^*a*^

Characteristic	HR	95% CI	*P*
Vascular invasion			
No	Reference		
Yes	2.58	1.58–4.21	**0**
Gender			
Female	Reference		
Male	1.77	0.95–3.3	0.074
Chemotherapy			
No	Reference		
Yes	2.69	1.09–6.65	**0.032**
AFP			
Normal	Reference		
Abnormal	0.16	0.04–0.65	**0.011**
HBV			
HBV−	Reference		
HBV+	1.95	1.31–2.89	**0.001**
PDW			
Normal	Reference		
Abnormal	1.5	0.95–2.37	0.083
Lymph node invasion			
No	Reference		
Yes	4.24	1.51–11.86	**0.006**
HBsAg			
Negative	Reference		
Positive	1.46	0.71–3	0.305
Tumor size			
<5 cm	Reference		
≥5 cm	2.82	1.74–4.57	**0**
Tumor number			
Single	Reference		
Multiple	1.38	0.69–2.78	0.365
Tumor differentiation			
High	Reference		
Low	1.11	0.7–1.74	0.666

^*a*^ Statistically significant *P*-values are in bold (*P *< 0.05).

**TABLE 5:** Multivariate Cox regression analysis for survival prediction on RFS^*a*^

Characteristic	HR	95% CI	*P*
Vascular invasion			
No	Reference		
Yes	1.5	0.83–2.79	0.17
HBV			
HBV−	Reference		
HBV+	1.6	0.98–2.61	0.06
Tumor size			
<5 cm	Reference		
≥5 cm	2.1	1.2–3.64	**0.0084**
Lymph node invasion			
No	Reference		
Yes	3	0.4–22.5	0.29
Chemotherapy			
No	Reference		
Yes	3.5	1.4–9.03	**0.0081**
AFP			
Normal	Reference		
Abnormal	0.18	0.042–0.78	**0.022**

^*a*^ Statistically significant *P*-values are in bold (*P *< 0.05).

## DISCUSSION

HCC is linked to microbiota. The relationship between the intratumoral microbiome profile and HCC development is still unknown. The tumor-resident intracellular microbiota is an innovative tumor component that has been identified in a number of cancer types, but its biological role is unknown. Exploring strategies that target these microbiota may thus have the potential to advance cancer therapy (14).

We conducted a large-scale analysis of the microbiota within the TME for liver cancer patients by examining tumor samples, adjacent tumor tissue, and normal tissue from 213 patients with primary liver cancer. We confirmed the presence of the microbiome within the tumors using FISH and characterized the microbial communities using 16S rDNA sequencing. Our analysis revealed distinct microbial patterns across liver cancer tumors, adjacent tumor tissues, and normal tissues, supporting the existence of unique microbiome signatures associated with liver cancer. Consistent with previous studies, we observed variations in the abundance of particular taxa between tumor and normal tissues. Specifically, the tumor-associated microbiota in HCC predominantly consists of genera such as *Bacteroides*, *Ochrobactrum*, *Akkermansia*, and *Lactobacillus*, with significant variability in their abundance across different samples.

Additionally, we aimed to elucidate the characteristics of hepatocarcinogenesis in liver cancer patients. While chronic HBV infection is a well-established risk factor for HCC, at least 20% of HCC cases are attributable to factors other than HBV or hepatitis C virus (HCV) infection (15). This underscores the necessity of investigating other potential bacterial factors that may contribute to the pathogenesis of HCC in the context of patients without a definitive history of HBV or HCV infection.

We therefore explored the microbiota in the HCC TME through large-scale analysis of tumor tissue samples from 166 HBV+ and HBV− patients. The analysis revealed a unique microbiological pattern between HBV+ and HBV− tumor tissues in HCC patients, suggesting that HBV typing enables significant clustering of HCC microbiome characteristics. In addition, we found differences in the abundance of specific taxa between tumor tissue from HBV+ and HBV− patients. The results showed that the tumor-related microbiota of HCC patients was mainly composed of *Bacteroides*, *Ochrobactrum*, *Akkermansia,* and *Lactobacillus*, and its abundance varied significantly among the different specimens. In addition, by integrating the overall microbial characteristics of the tumor samples, the relative abundance of taxa at the genus level was aggregated to determine whether the patients were infected with HBV. The results show that when considering vascular invasion, sex, chemotherapy, AFP, PDW, lymph node invasion, HBsAg, tumor size, tumor number, tumor differentiation, and other previously common prognostic factors, HBV has significant prognostic value. High levels of *Bacteroides* and low levels of *Myxobacterium*_AT3-03 and *Lachnospiraceae_bacterum*_609 correlate with improved good OS and RFS.

Compared with recent studies (16), our research presents new insights and holds potential translational value. First, by analyzing a large cohort, we revealed the significant association between the microbiota within liver cancer tumors and patient prognosis. Additionally, our findings suggest that the microbiome in HCC tumors is associated with HBV infection, supporting the correlation between specific bacterial taxa and HBV infection, a finding that is supported by other studies (31). These insights offer fresh perspectives for clinical translational research into the role of the microbiome in HCC tumors. Second, our study underscores the heterogeneity of the microbiome across different HCC tumors and within individual tumors. The microbiota distribution within a tumor is not random but is structured into microhabitats that support immunological and epithelial cell functions, thereby promoting cancer growth—observations corroborated by recent research (21). The extent to which this intratumoral microbiome heterogeneity affects HCC progression and patient outcomes warrants further investigation.

According to earlier research, there are three primary ways in which the microbiome within the tumor interacts with the host (22): (i) directly influencing tumorigenesis through DNA damage; (ii) regulating carcinogenic signaling pathways; and (iii) controlling the inflammatory response and host immune system. The impact of the microbiome within the tumor on the prognosis of HCC patients is not well understood. Further analysis of the livers of HBV+ and HBV− patients revealed that the former had a worse prognosis and was marked by a high abundance and diversity of *Bacteroides*. These results are in line with earlier research showing that *Bacteroides* are present in HCC (16). Studies indicate that short-chain fatty acids (SCFAs), such as butyric acid, propionic acid, and acetic acid, are produced when *Bacteroidetes* ferment dietary fiber in the gut. These SCFAs influence host metabolism in a number of ways by acting on G protein-coupled receptors expressed by intestinal endocrine cells (24). Enrichment was detected in the tumor tissue and gut microbiota of previously HBV+ patients (21). A key species of *Akkermansia* is *Bacillus myxophilus*, a mucin-degrading bacterium that has been linked to a decreased risk of obesity. This species also has multiple metabolic functions and maintains the integrity of the gastrointestinal mucosa (23, 32). *Lactobacillus* is enriched in HCC patients. Probiotics such as *Lactobacillus*, which is widely used, have been shown to be beneficial for treating gastrointestinal disorders, including diarrhea, nonalcoholic fatty liver disease, inflammatory bowel disease, and gastrointestinal infections (17). These bacteria all have a similar role in metabolism.

This research topic still has certain limitations. The microbiome is also affected by confounding factors. Broad-spectrum antibiotics significantly reduce the diversity of the microbiota; a high-fat diet promotes the proliferation of bile acid-metabolizing bacteria; advanced tumors have more severe immune suppression and microbiota alterations; and the colonization patterns of region-specific strains are different. In this project, we used the FISH method to confirm the presence of bacteria, and many other studies have also applied this method to verify the existence of bacteria. However, in this project, we did not use the culture method to determine the survival status or activity of bacteria in tumor tissues, although some studies have successfully cultured some bacteria. I believe there are several reasons why bacteria in tissues are difficult to culture, reasons related to the bacteria themselves: (i) bacteria are sensitive to oxygen, and conventional culture conditions lead to their death; (ii) they rely on specific metabolites from the host or products from symbiotic bacteria; (iii) they have a low abundance in tissues and a long doubling time. Traditional culture media cannot simulate the microenvironment of tissues, and the culture time is short. Some bacteria require growth factors provided by other bacteria, and a single culture medium cannot meet these requirements.

HBV genotypes play a significant role in the pathogenesis and disease progression. However, the relationship between HBV genotypes and bacteria was not explored in this study. In China, the predominant HBV genotypes are B and C. Genotype B is often associated with mild liver injury, while genotype C is the predominant genotype in Asia; it is more prone to severe conditions, liver cirrhosis, and liver cancer and has a higher risk of liver cancer. It is generally believed that genotype B, which is more common in southern China, is less virulent than genotype C, which is more common in northern China. Recently, it has been reported that a novel recombinant hepatitis B virus between genotypes B and C has a higher viral load (33).

Many *Bacteroides* have been found in the gut microbiome of patients with colorectal cancer. Previous studies have shown that an increase in the number of *Bacteroides* groups in the gut may shorten patients’ lifespans once cancer begins and increase their likelihood of developing cachexia (34). Patients with HBV+ HCC had increased levels of *Bacteroidetes*. According to our research, a positive prognosis is linked to high levels of *Bacteroidetes* in HCC tissues, indicating that *Bacteroidetes* may also be helpful in the HCC TME. Furthermore, our data showed a significant correlation between *Akkermansia* and *Bacteroides* abundance.

Metabolic changes may be the most significant factors influencing advanced malignancy and unfavorable clinical outcomes. Targeting these metabolic pathways is a promising treatment approach for HCC (35). Metabolic changes may be the most important factor affecting advanced malignant tumors and adverse clinical outcomes. Targeting these metabolic pathways is a promising approach for treating hepatocellular carcinoma. There are often severe hypoxic areas (oxygen partial pressure <5 mmHg) within solid tumors due to vascular abnormalities, which provide a survival advantage for both obligate and facultative anaerobic bacteria. Bacteria can enter the tumor through blood flow, adjacent tissues, or immune cells and colonize in hypoxic areas (36). Aerobic bacteria and anaerobic bacteria were found in hepatitis B virus-positive HCC samples. The novel oxygen-tolerant phenotype of these bacteria in cancer patients may be an important aspect of metabolic changes. Anaerobic bacteria break down glucose or other carbon sources through anaerobic fermentation, producing SCFAs (such as butyric acid and propionic acid), lactic acid, hydrogen, and other metabolites. SCFAs such as butyric acid can be taken up by tumor cells as an energy source, promoting their proliferation (37). Lactic acid further acidifies the microenvironment, enhancing tumor invasiveness. Some bacteria, such as *Fusobacterium nucleatum*, can break down host proteins and release amino acids (such as aspartic acid), providing raw materials for tumor cells to synthesize nucleotides and accelerate their growth.

It is important to take into account several possible study limitations. Initially, the random forest model should be assessed in multicenter research, as it was created and validated in a single-center cohort study, which restricts the applicability of our findings. Second, the study population consisted of individuals with HBV-related HCC, which restricts the application of the findings to HCC patients. Last, the study’s retrospective design and the absence of healthy volunteers for comparison severely restrict the study’s findings.

### Conclusion

In conclusion, our analysis provides new insights into the microbiome profile of both primary liver cancer and HCC patients, revealing how specific taxa correlate with HCC clinical features and that the abundance of the intratumoral microbiota may affect HCC patient survival. HBV infection has also been found to significantly affect the postoperative survival of HCC patients, with HBV+ patients having a poorer prognosis than HBV− patients. Therefore, detecting whether a patient is infected with HBV can help predict the prognosis of HCC patients to a certain extent. Furthermore, it has been demonstrated that HBV+ has some effect on HCC metabolism; hence, our study adds biological information to the understanding of the clinical characteristics of HCC.

## Data Availability

The raw data supporting the conclusions of this study are available in the NCBI database under BioProject no. PRJNA1125920.

## SUPPLEMENTAL MATERIAL

Supplemental material spectrum.01188-25-s0001.docx Fig. S1 and S2; Tables S1 and S2.

ASM does not own the copyrights to Supplemental Material that may be linked to, or accessed through, an article. The authors have granted ASM a non-exclusive, world-wide license to publish the Supplemental Material files. Please contact the corresponding author directly for reuse.
