# Supplementary material for: Identification of intratumoral bacteria correlated with hepatitis B virus (HBV) levels: a prognostic indicator for patient outcomes in hepatocellular carcinoma patients
Source: Microbiol Spectr. 2025 Oct 23;13(12):e01188-25. doi: 10.1128/spectrum.01188-25 (PMC12671140; doi:10.1128/spectrum.01188-25)
Supplement: Supplemental material — Fig. S1 and S2; Tables S1 and S2. [file spectrum.01188-25-s0001.docx]

**Identification of Intratumoral Bacteria Correlated with Hepatitis B Virus (HBV) Levels: A Prognostic Indicator for Patient Outcomes in Hepatocellular Carcinoma**

Yuan Dang^1,2, †^, Jingyun Huang^1,2, †^, Xing Peng^3^, Yingchao Wang^4^, Jianmin Wang^1,2, *^

1 Innovation Center for Cancer Research, Clinical Oncology School of Fujian Medical University, Fujian Cancer Hospital, 420 Road Fuma, Fuzhou 350014, Fujian, China

2 Fujian Key Laboratory of Advanced Technology for Cancer Screening and Early Diagnosis, Clinical Oncology School of Fujian Medical University, Fujian Cancer Hospital, Fuzhou 350014, China

3 College of Computer and Data Science, Fuzhou University, Fuzhou 350108, China

4 The United Innovation of Mengchao Hepatobiliary Technology Key Laboratory of Fujian Province, Mengchao Hepatobiliary Hospital of Fujian Medical University, Fuzhou 350025, China

^†^These authors contributed equally to this work.

***Corresponding authors**

Jian-min Wang, Email: [wangjm8605@163.com](mailto:wangjm8605@163.com), Innovation Center for Cancer Research, Clinical Oncology School of Fujian Medical University, Fujian Cancer Hospital.

Supplementary Table 1…………………………………………………………………………... …2

Supplementary Table 2…………………………………………………………………………... …2

Supplementary Figure 1…………………………………………………………………....….…….4

Supplementary Figure 2……………………………………………………….……….………….11

**Supplementary Table 1.** Univariate Cox regression analysis for survival prediction on OS.

**
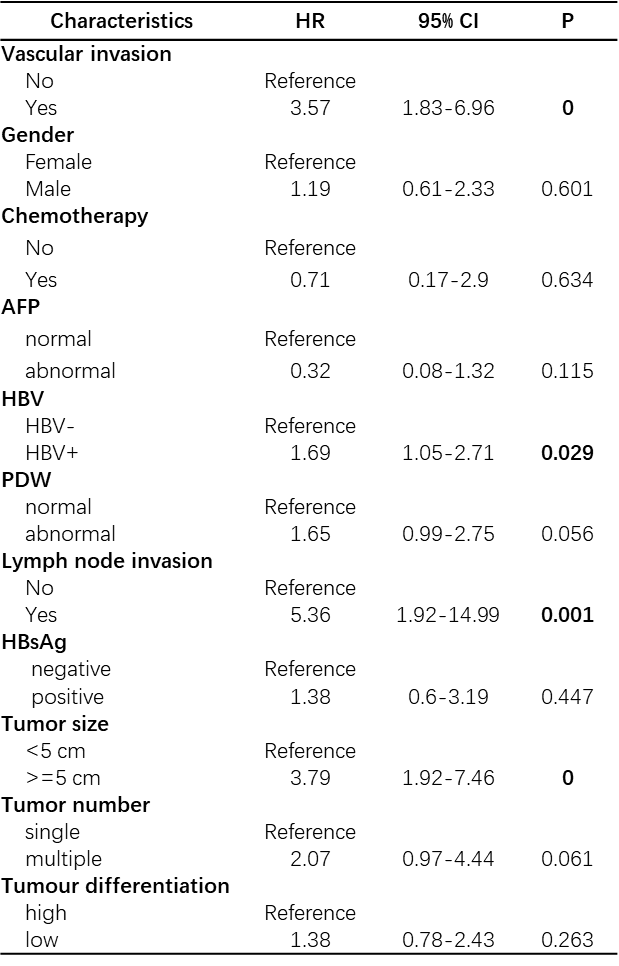
**

**Supplementary Table 2.** Multivariate Cox regression analysis for survival prediction on OS.


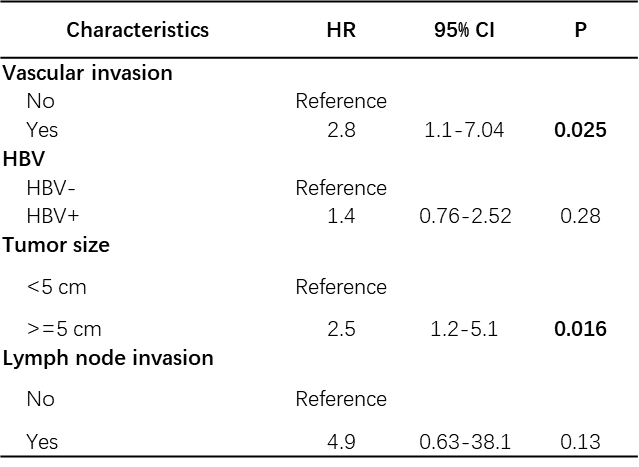


HR, hazard ratio; 95% CI, 95% confidence interval; P, statistically significant p-values are in bold (p<0.05).

**（A）**


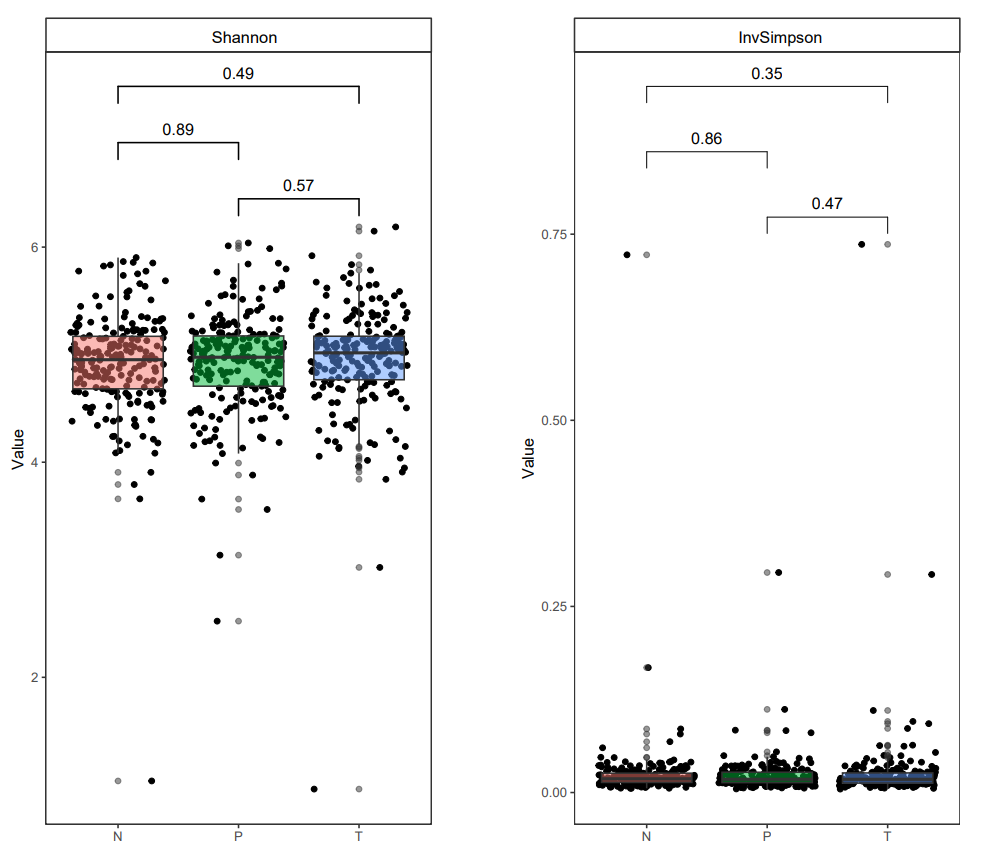


**（B）**

**（C）**


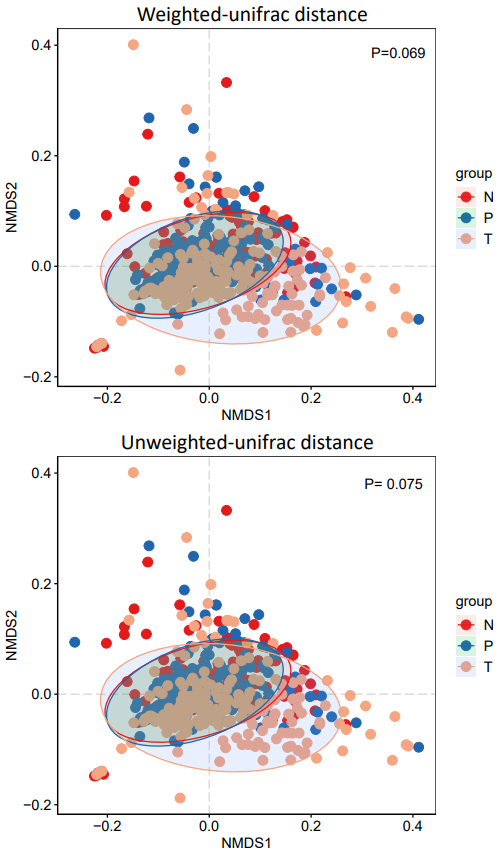

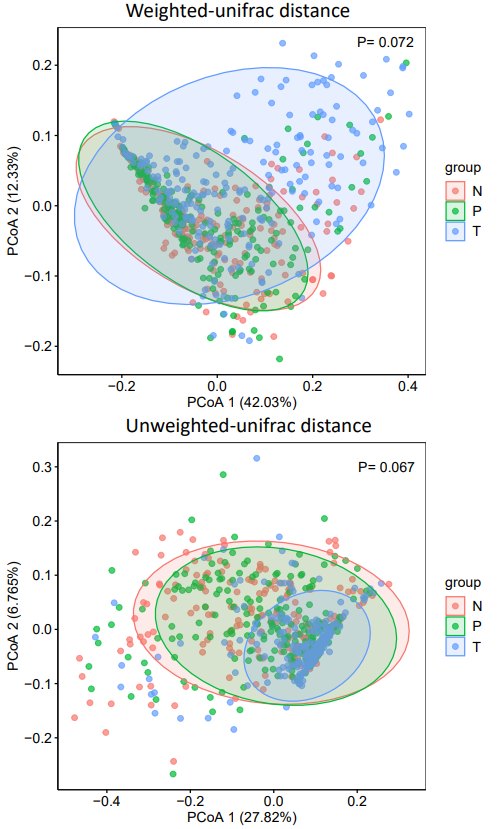


**(D)**


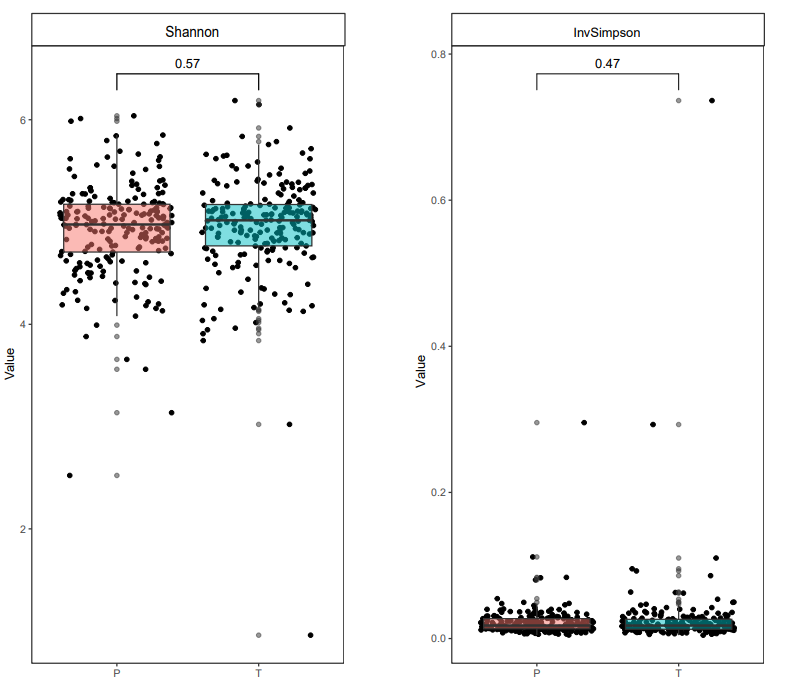


**(E)**

**(F)**


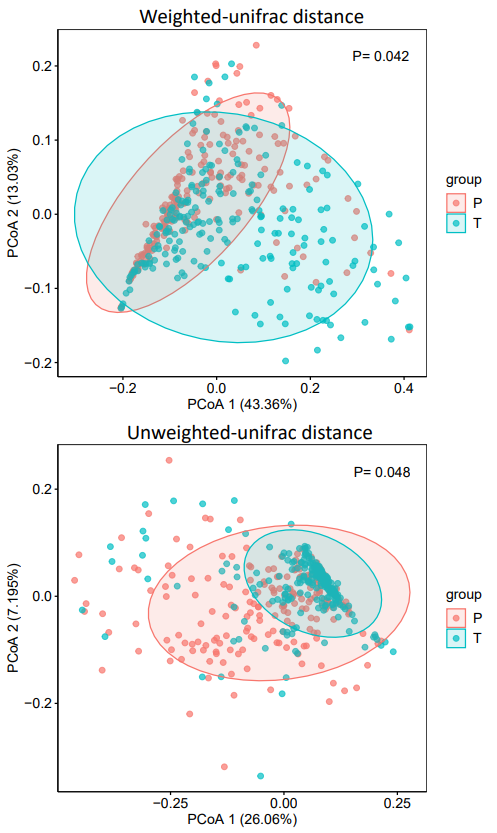

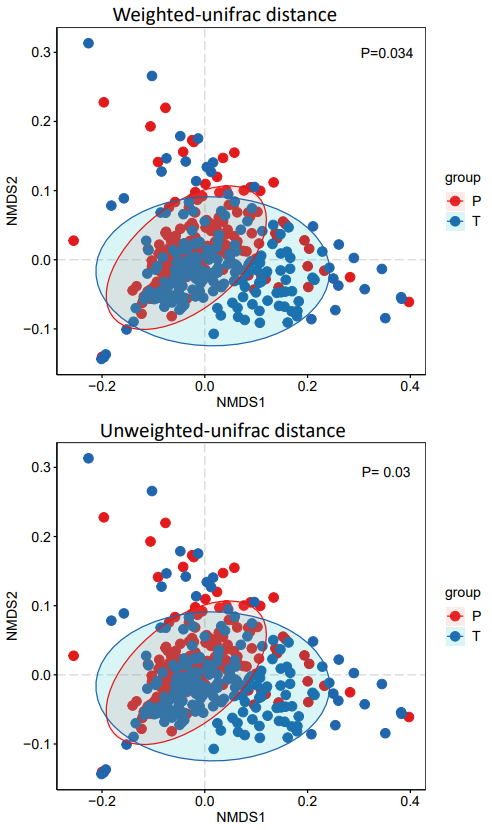


**(G)**


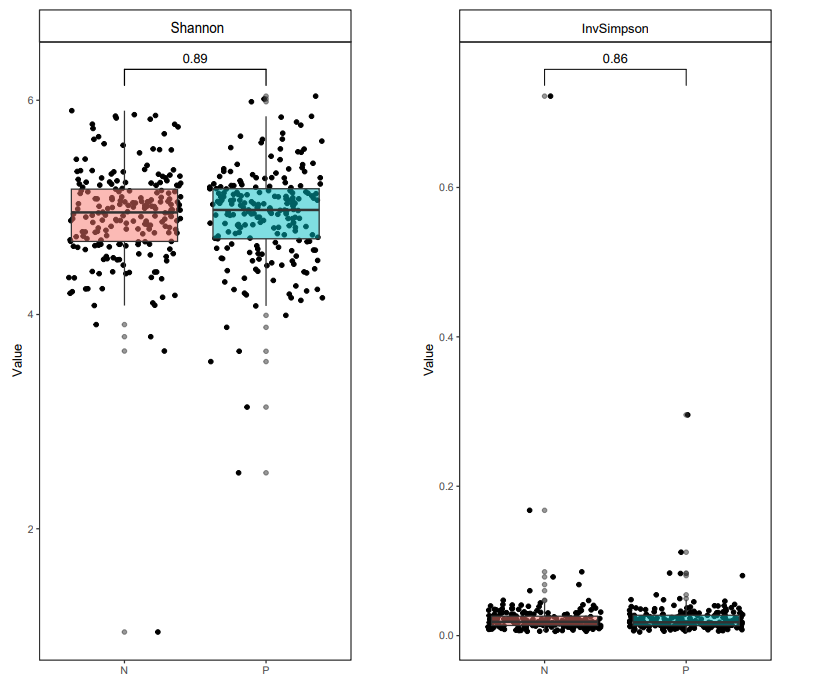


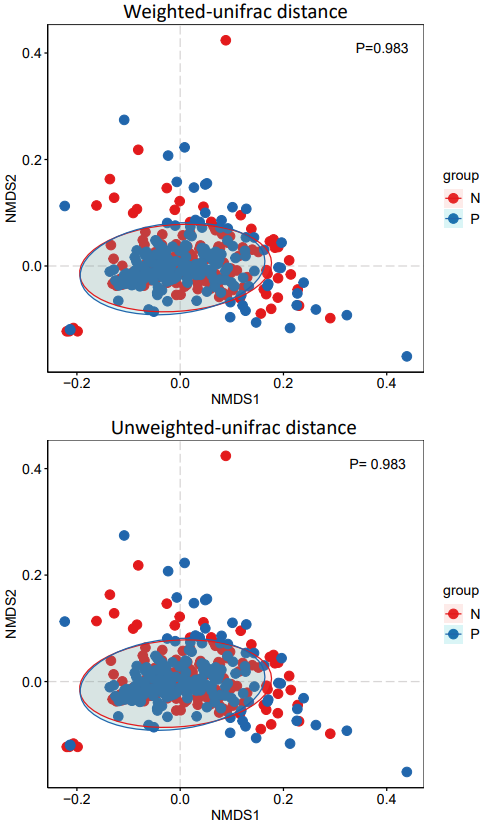

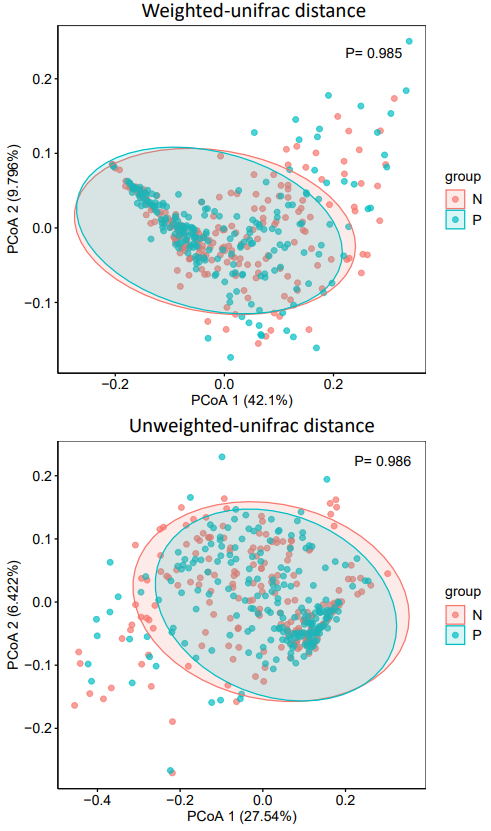


**(I)**

**(H)**

**(J)**


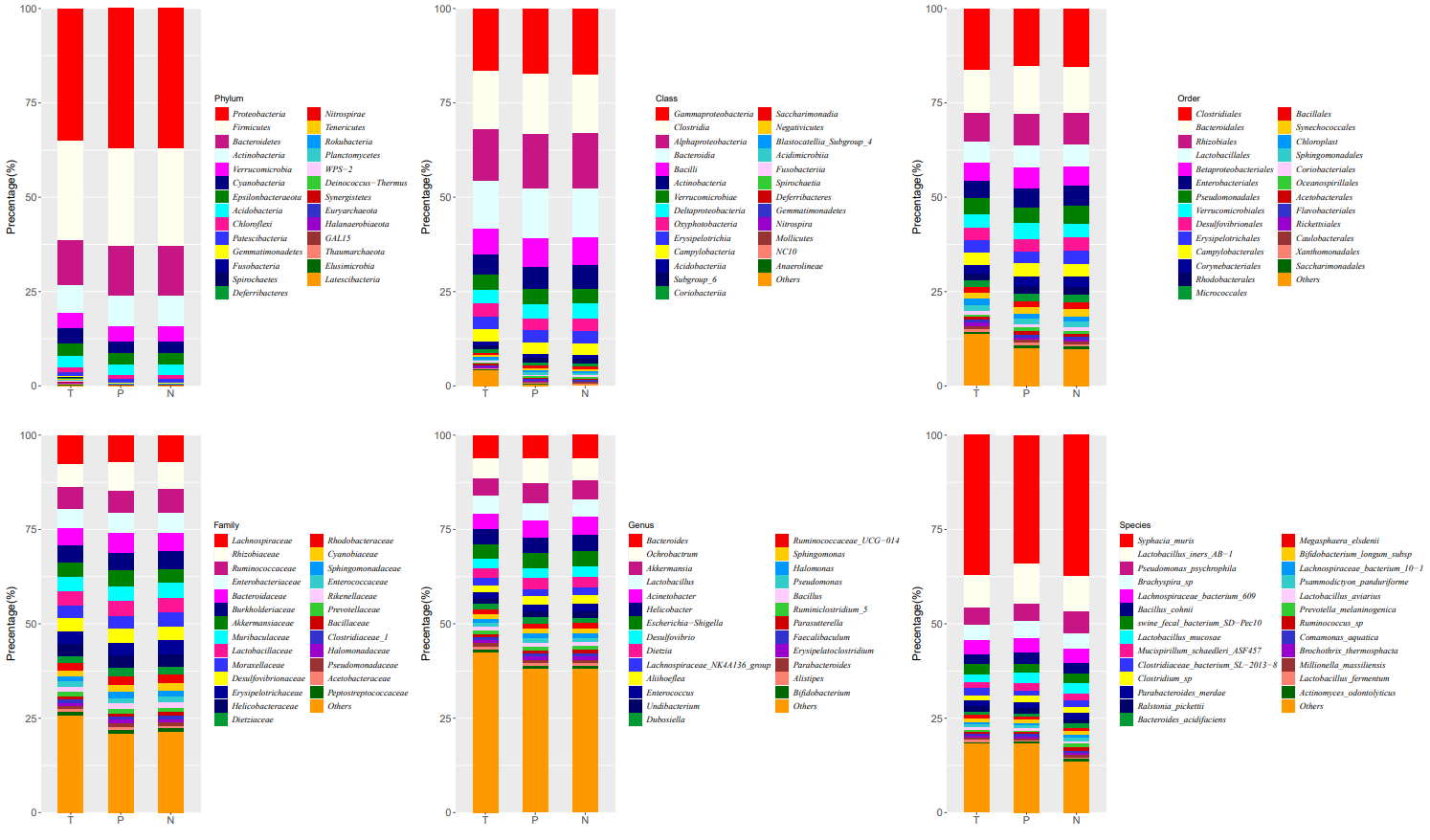


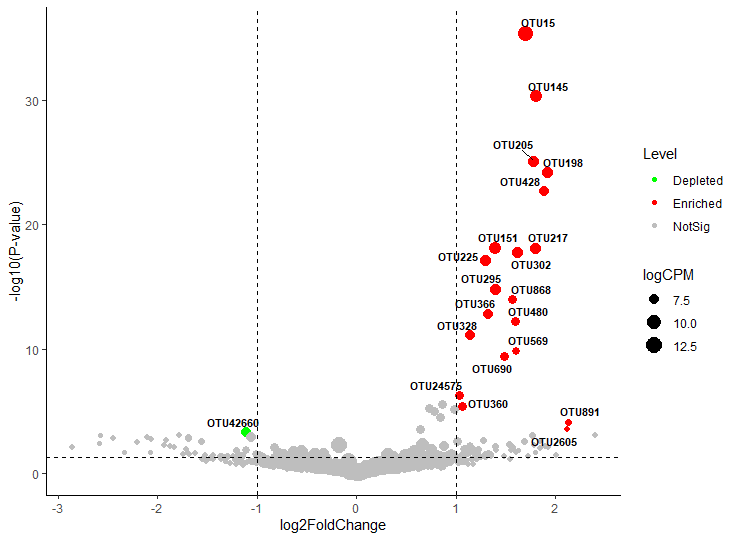

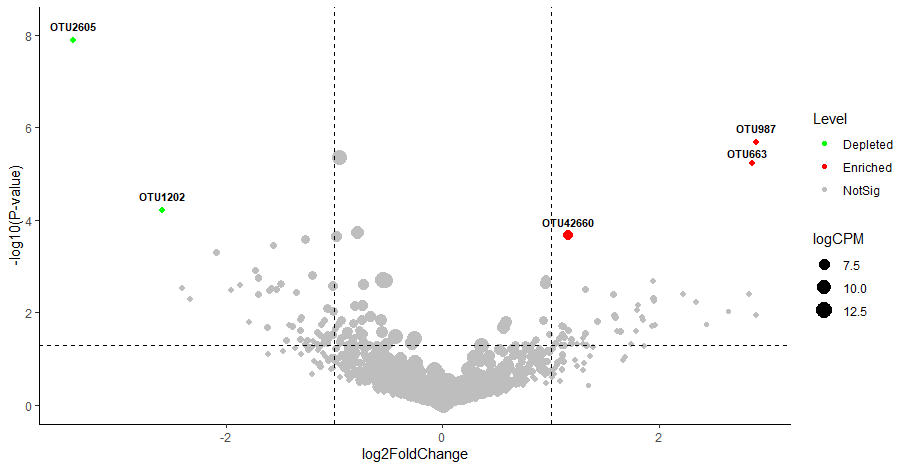


**(L)**

**(K)**

P vs N

T vs P

**（N）**

**(M)**


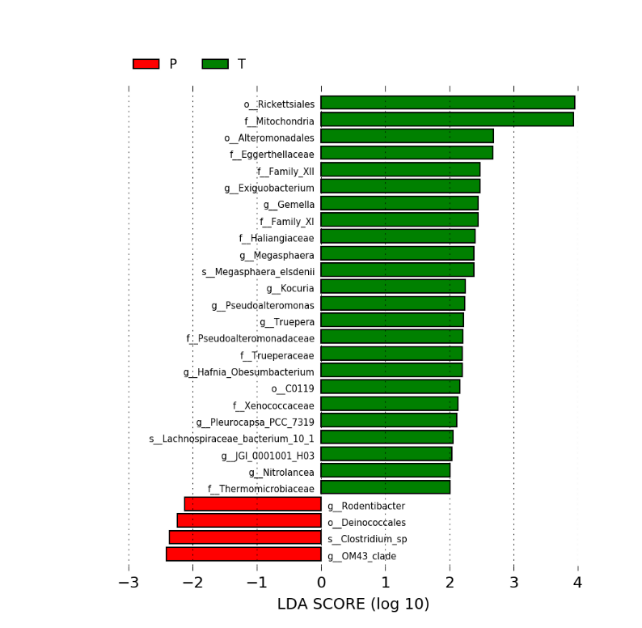

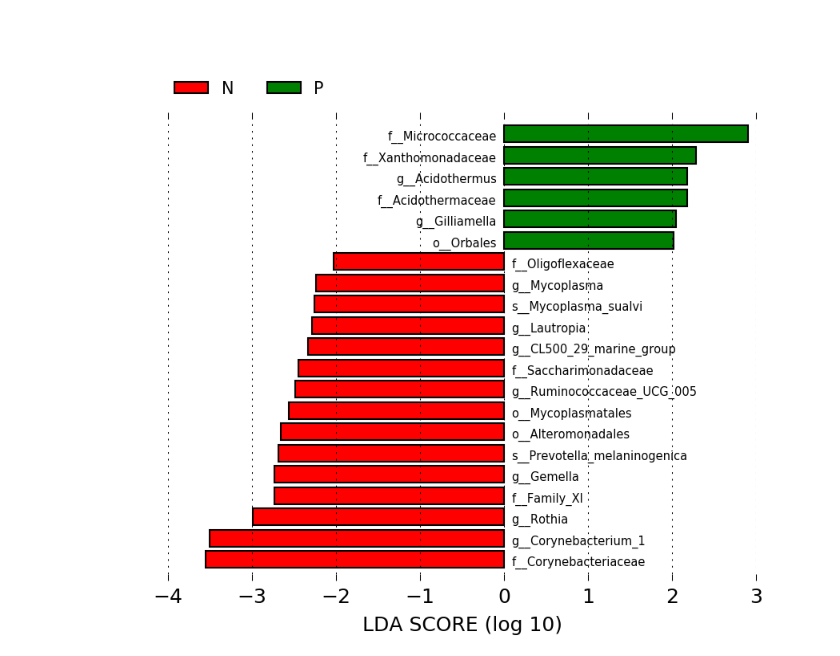


**(O)**


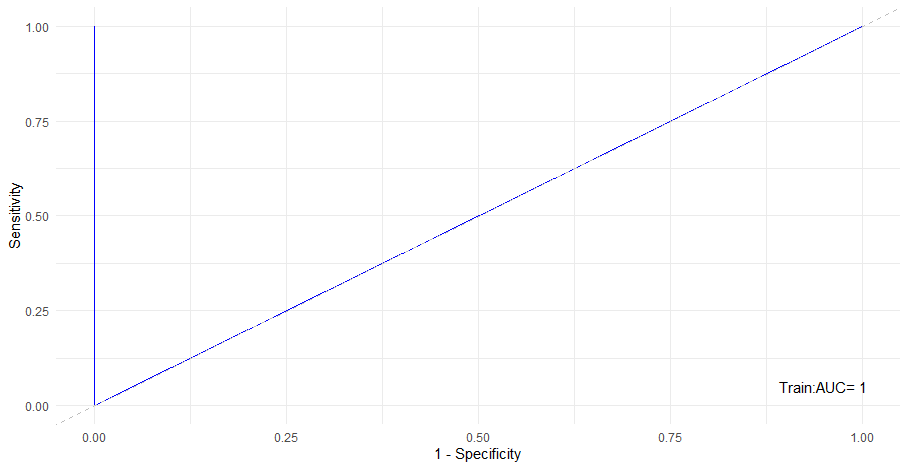


**(P)**


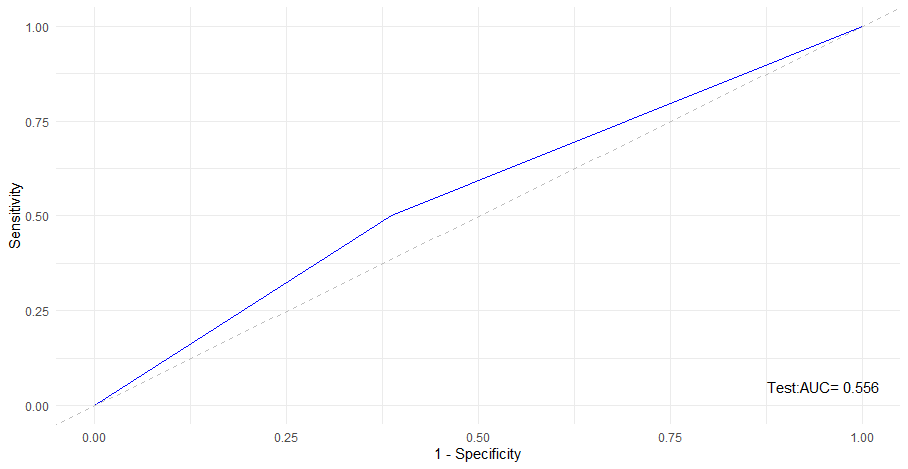


**（Q）**


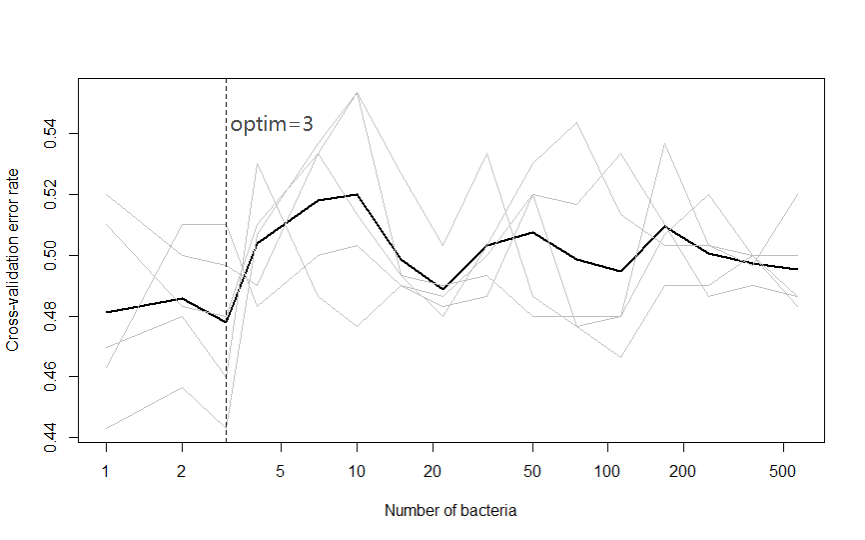


**Supplementary Figure 1.** 16S rDNA sequencing was performed on tumor tissues, adjacent tumor tissues, and normal tissues of 213 previously collected patients with primary liver cancer.

(A) Alpha diversity of tumor tissues, adjacent tumor tissues, and normal tissues. (B, C) β-diversity of tumor tissue, adjacent tumor tissue, and normal tissue. (D) α-diversity of tumor tissue and adjacent tumor tissue. (E, F) β-diversity between tumor tissue and adjacent tumor tissue. (G) α-diversity between normal tissue and adjacent tumor tissue. (H, I) β-diversity between normal tissue and adjacent tumor tissue. (J) Differences in the dominant bacterial communities (top 27 taxa in relative abundance) at the level of phylum, order, order, family, genus, and species in tumor, adjacent tumor, and normal tissues of patients with hepatocellular carcinoma. (K) Volcano plot of the abundance of different OTUs in normal tissue versus adjacent tumor tissue. The cut-off condition is (|log2 fold-change| > 1, p < 0.05), and OTUs that are significantly reduced in the tissue are shown in green; OTUs that are significantly enriched in the tissue are shown in red. (L) Volcano plot of the abundance of different OTUs in tumor tissues and adjacent tumor tissues. The cut-off condition is (|log2 fold-change| > 1, p < 0.05), OTUs that are significantly reduced in the tissue are shown in green; OTUs that are significantly enriched in the tissue are shown in red. (M) Linear discriminant analysis effect size (Lefse) analysis of neighboring tumor tissues versus tumor tissues, and linear discriminant analysis (LDA) histograms of taxa with differences in abundance. Only LDA SCORE (Log 10) >2.0 and P<0.05 were characterized. (N) Linear discriminant analysis effect size (Lefse) analysis of normal tissues versus adjacent tumor tissues, linear discriminant analysis (LDA) histograms of taxa with differences in abundance. Only LDA SCORE (Log 10) >2.0 and P<0.05 were characterized. (O) AUC plot of the predictive effect of the random forest model built by genus colony on the training set. (P) AUC of the predictive effect of the random forest model built by genus flora on the test set.(Q) The general accuracy of the three genus microbial features in distinguishing between tumors and normal tissues was 52.21 % and the error rate was 47.79 %.

**(A)**


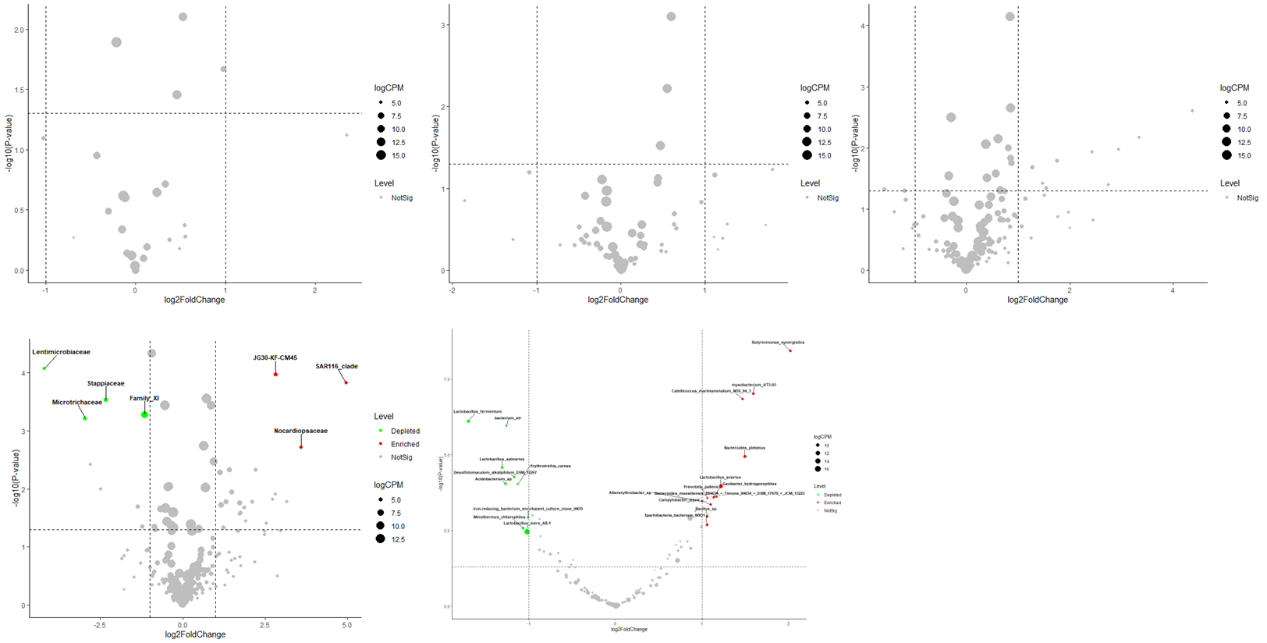


**order**

**phylum**

**class**

**family**

**species**


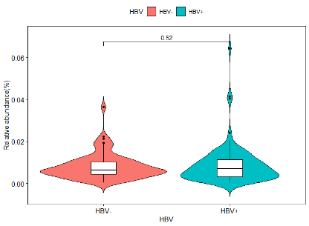

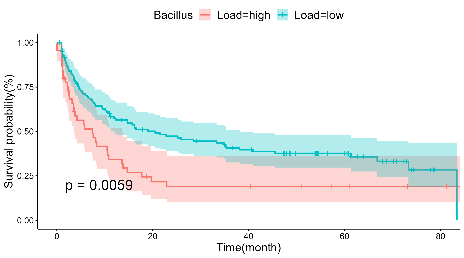

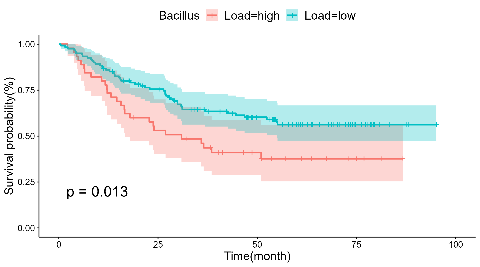


**(B)**


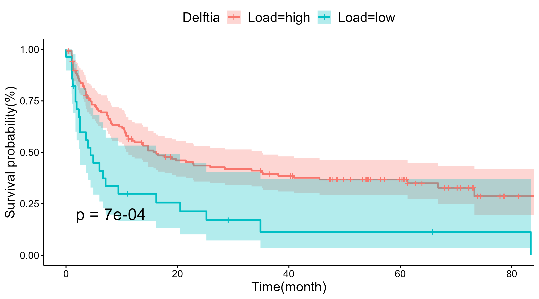

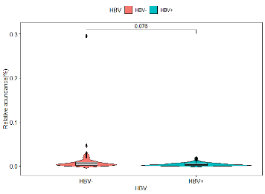

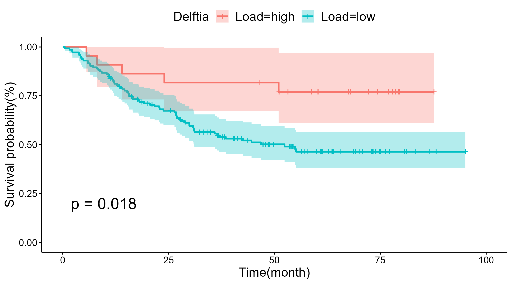


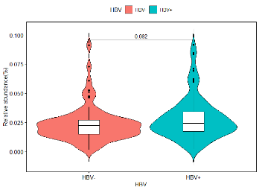

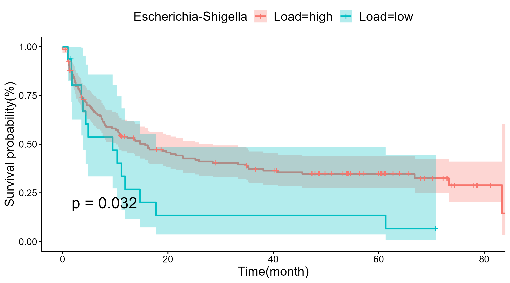

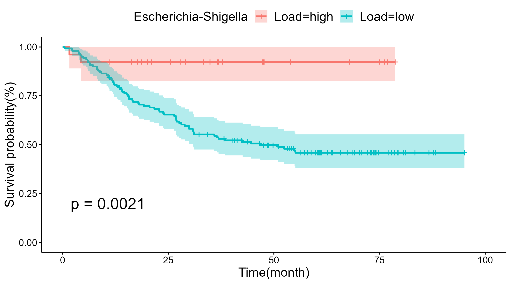


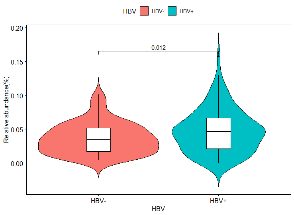

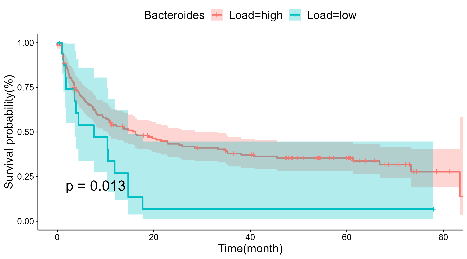

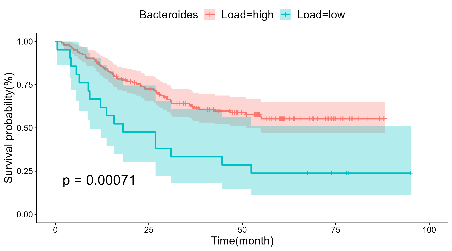


**(C)**


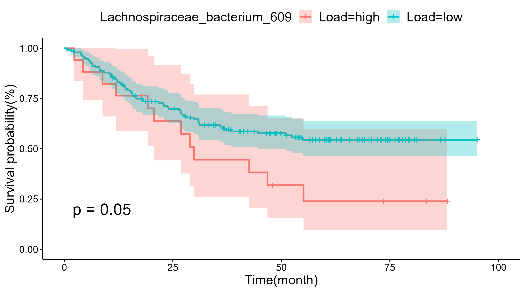

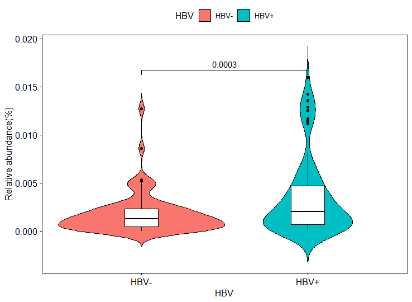

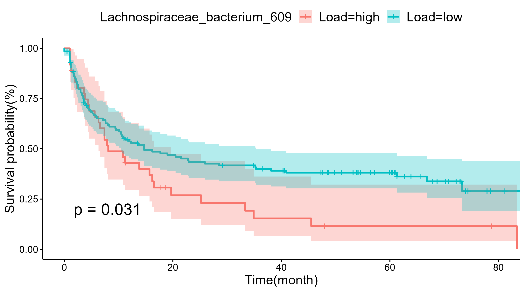


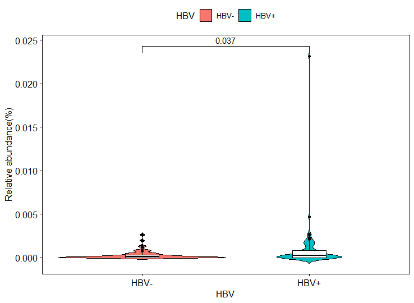


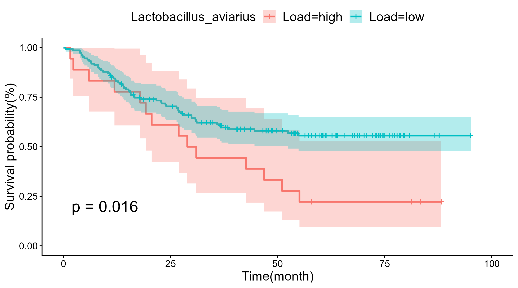

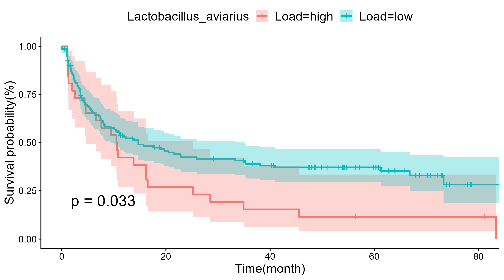


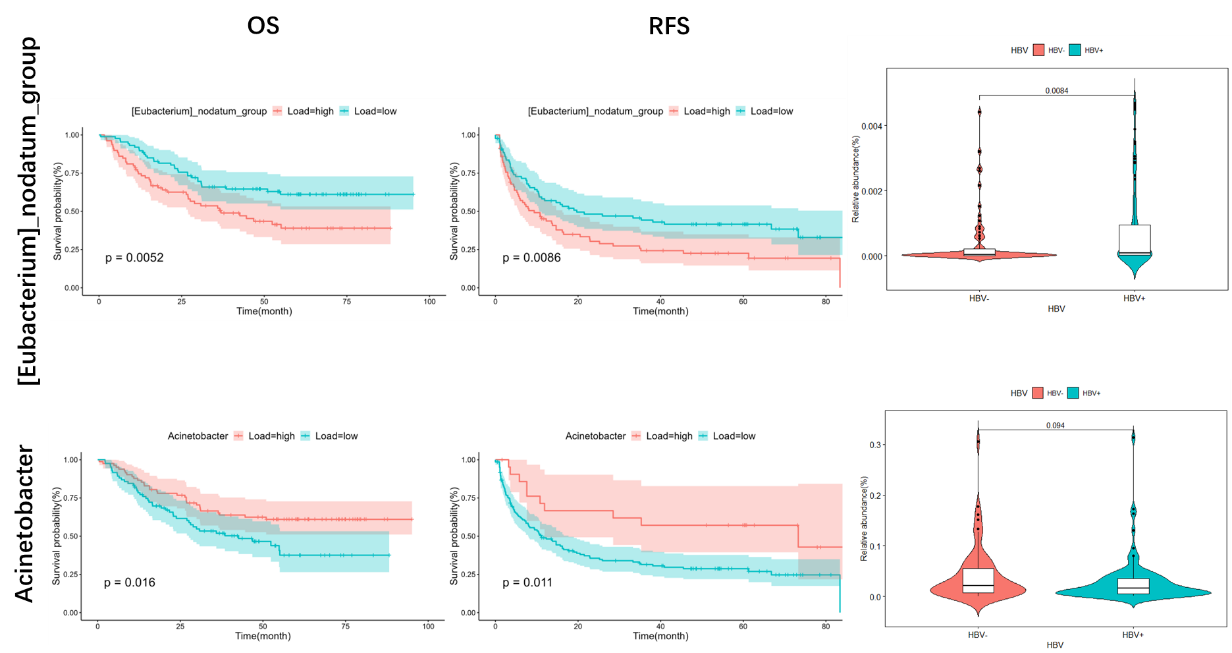


**(D)**


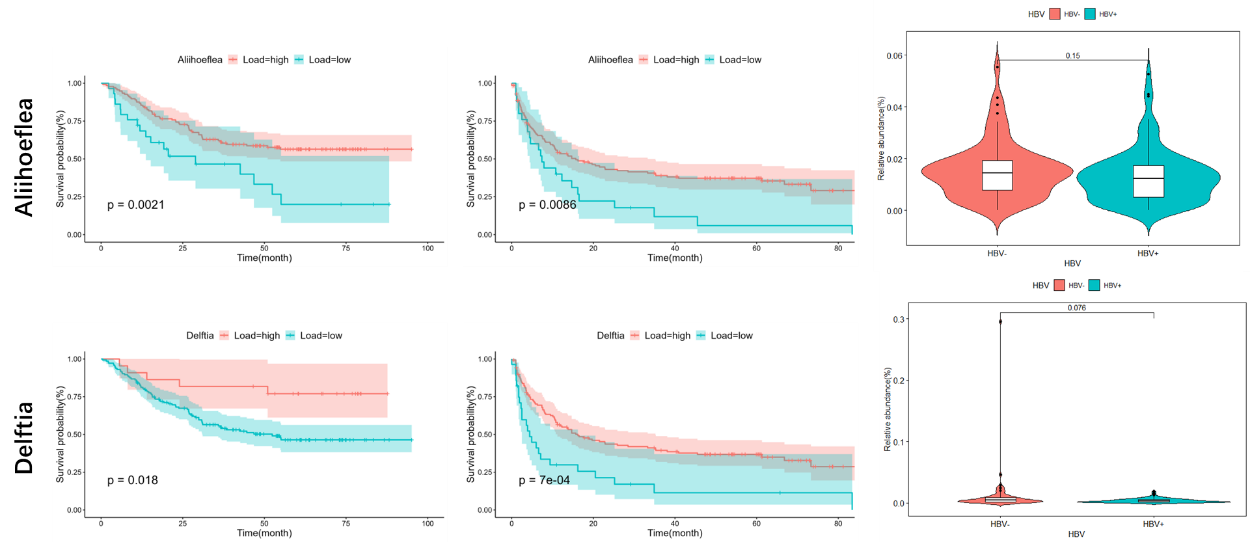


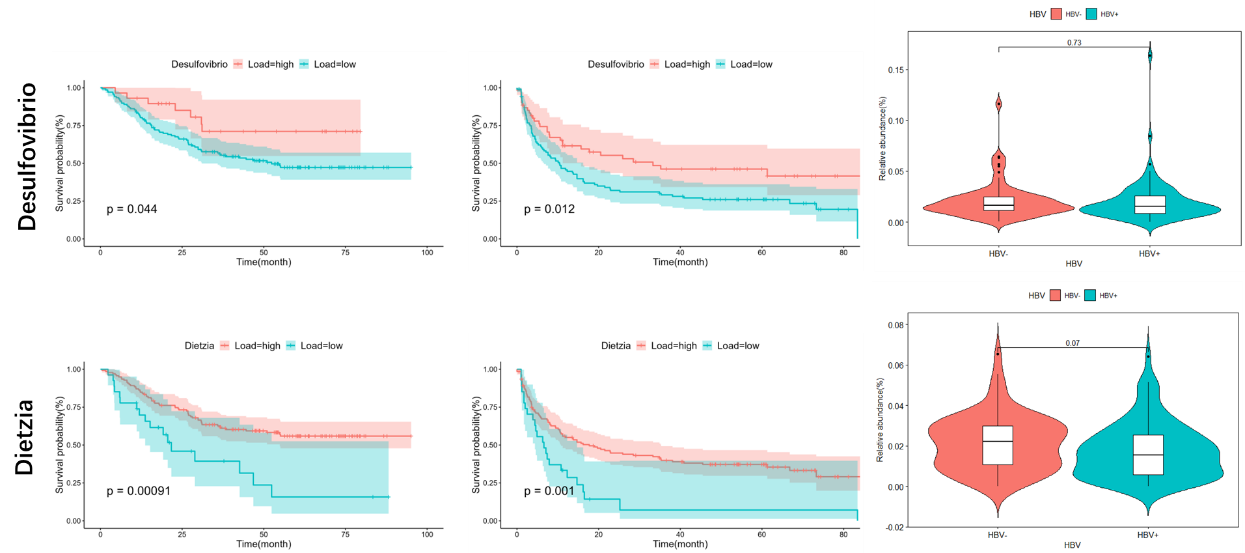


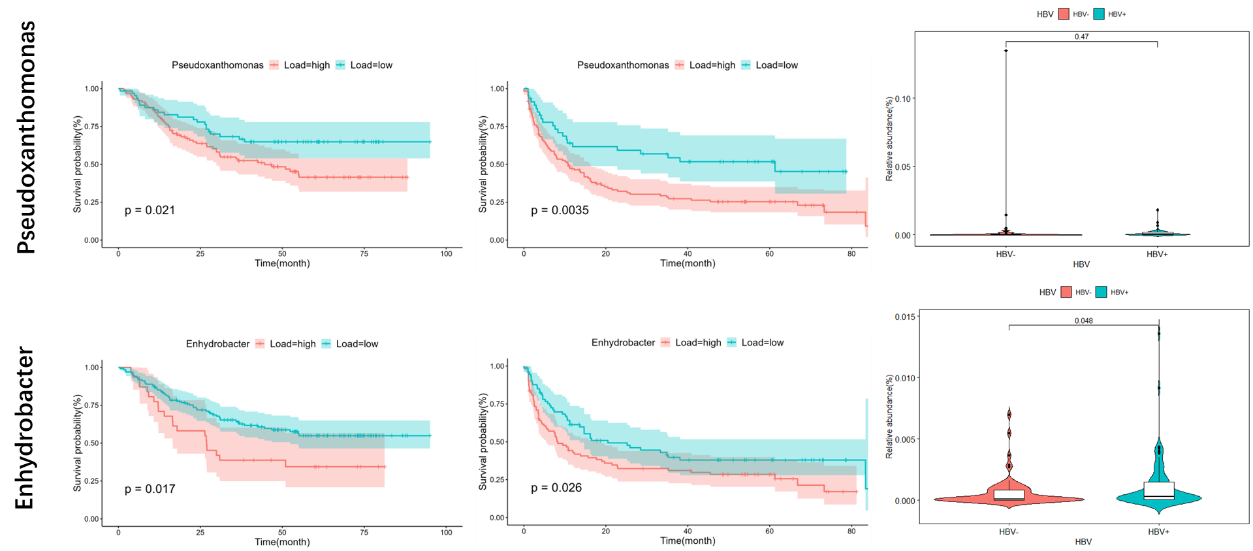


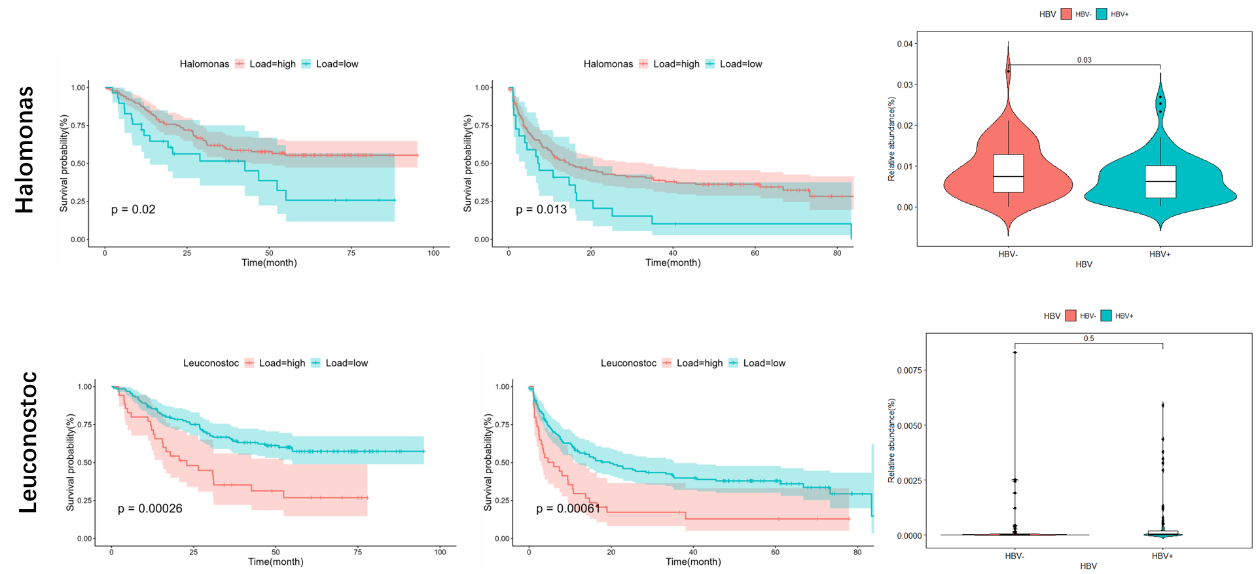


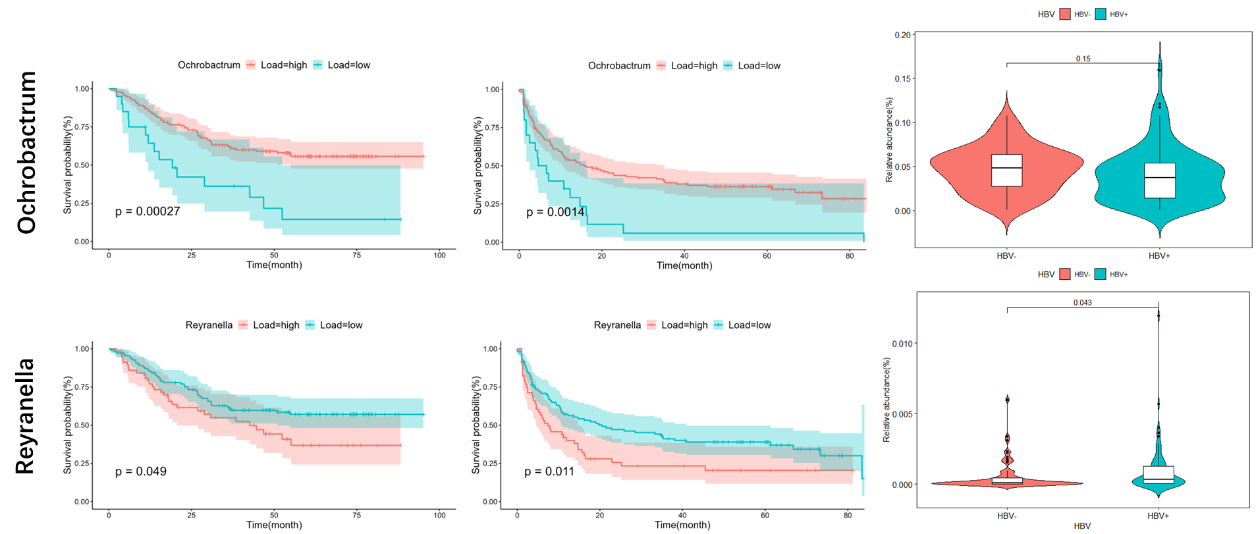


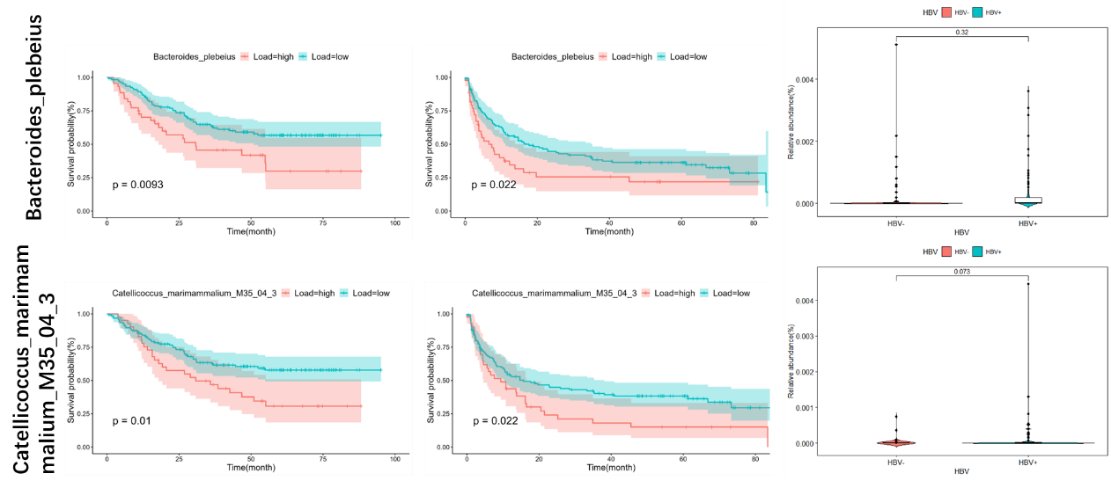


**(E)**


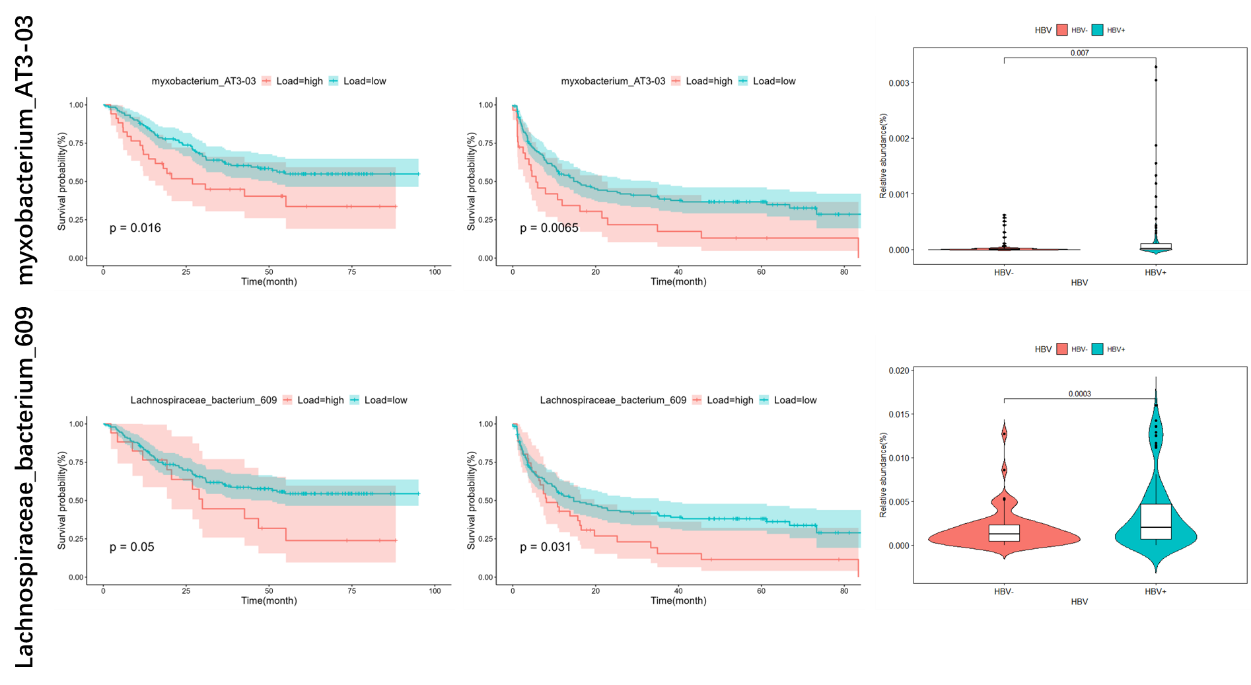


**(F)**


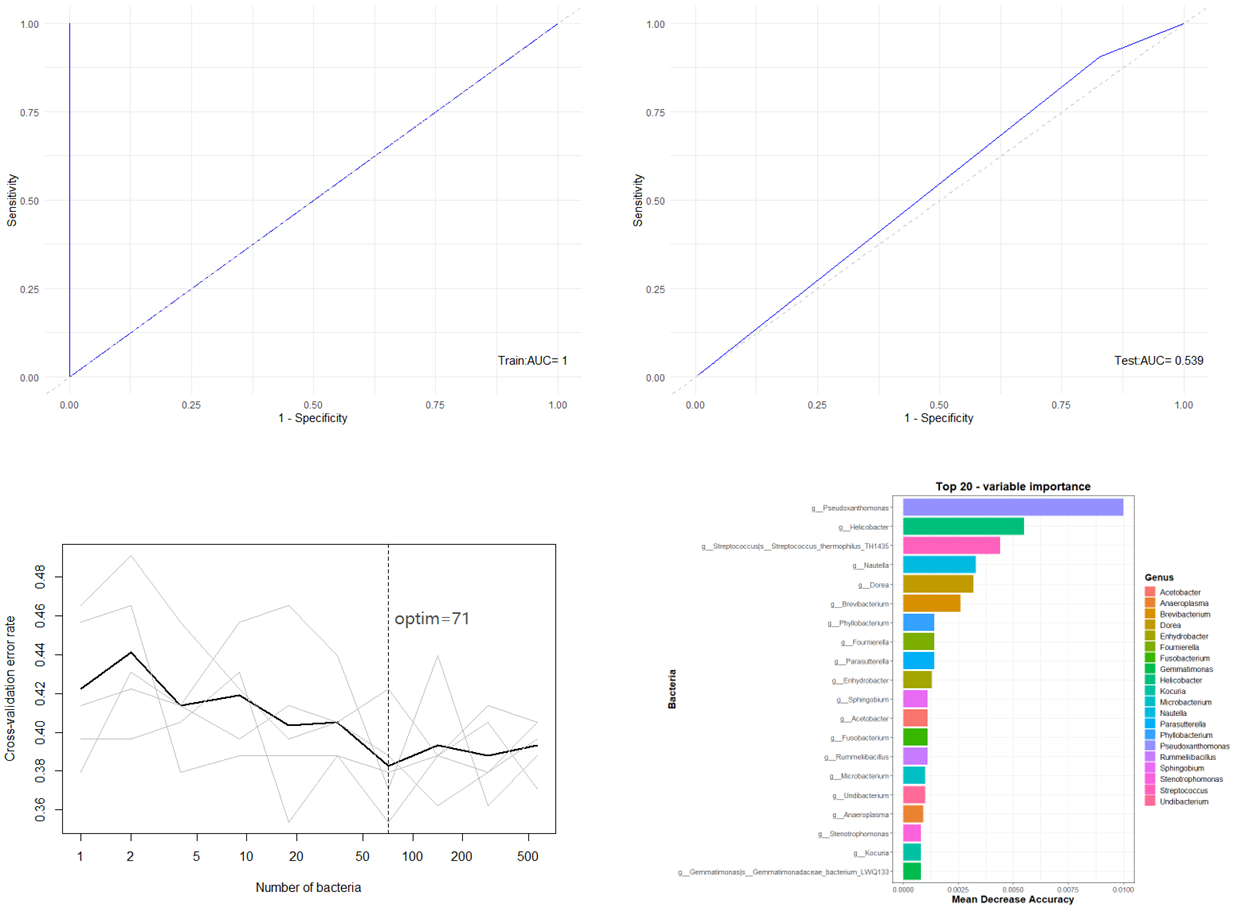


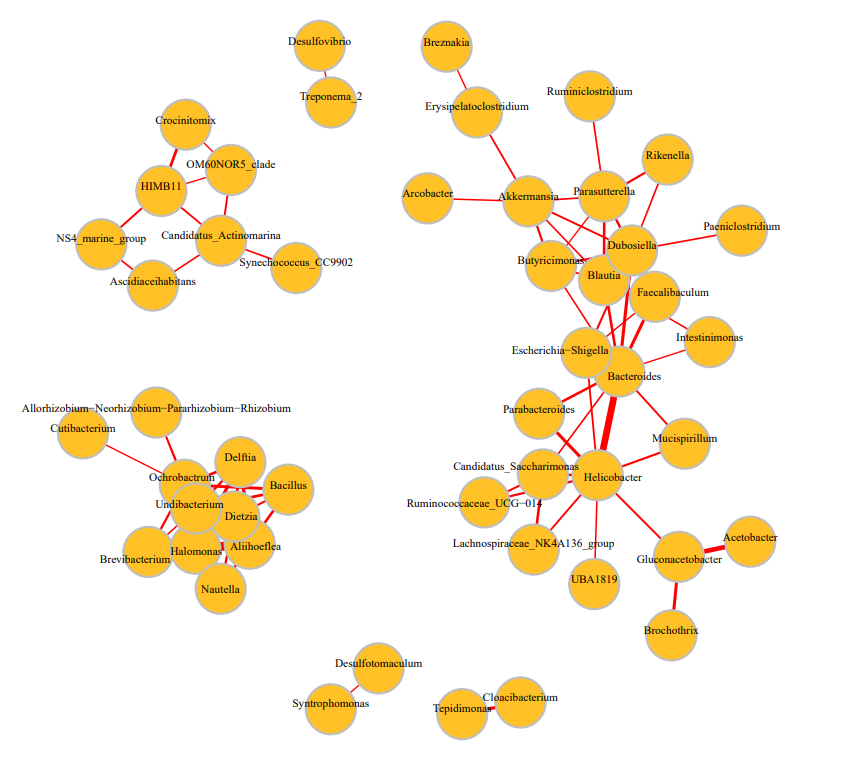


**(G)**


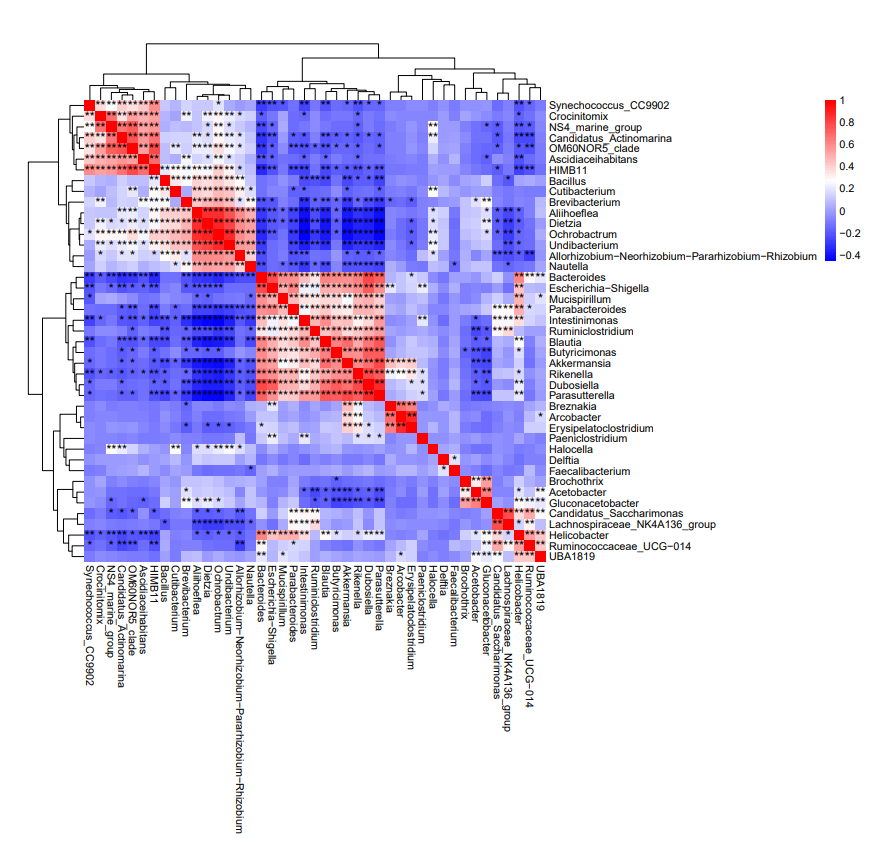

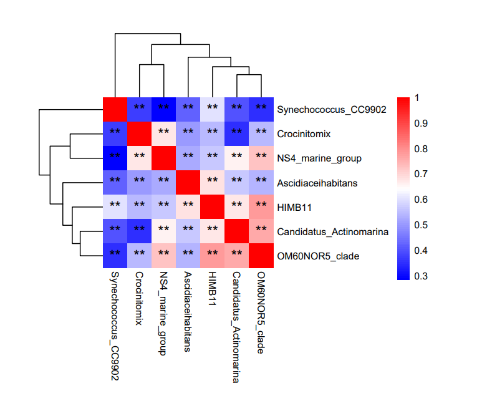


**(H)**

**Supplementary Figure 2.**16S rDNA sequencing was performed on 166 previously collected tumor tissues from HBV+ and HBV- patients with hepatocellular carcinoma (HCC).

(A) Volcano plot of microbial abundance of phylum, order, family, and species in tumor tissues of HBV+ versus HBV- patients. The cutoff condition is (|log2 fold-change| > 1, p < 0.05), and bacteria that are significantly reduced in the tissue are shown in green; bacteria that are significantly enriched in the tissue are shown in red. (B) DESeq analysis of the survival curves of bacteria with higher relative abundance of genus levels in tumor tissues of HBV+ versus HBV- patients with differences in both OS and RFS and their relative abundance violin plots in tumor tissues of HBV- and HBV+ patients. Comparison of relative abundance between groups was performed by t-test. (C) DESeq analysis of the survival curves of bacteria with higher relative abundance of species levels in tumor tissues of HBV+ versus HBV- patients differing in both OS and RFS as well as their relative abundance violin plots in tumor tissues of HBV- and HBV+ patients. Comparison of relative abundance between groups was performed by t-test. (D) Lefse analysis Survival curves of bacteria differing in genus-level abundance in tumor tissues of HBV+ and HBV- patients at both OS and RFS and violin plots of their relative abundance in tumor tissues of HBV- and HBV+ patients. Comparison of relative abundance between groups was performed using t-test. (E) Lefse analysis of the survival curves of bacteria with differences in species-level abundance in tumor tissues of HBV+ and HBV- patients that differed in both OS and RFS as well as their relative abundance violin plots in tumor tissues of HBV- and HBV+ patients. Comparison of relative abundance between groups was performed by t-test. (F) AUC curves for the training and testing of the random forest model and the calculated general accuracy of 61.72 % and error rate of 38.28 % for the 27 generic colony features in distinguishing between differentiating HBV- and HBV+ patients, as well as bar graphs of the top 20 important features of the generic colony . (G) Network plot of correlation between genus-level bacteria in tumor tissues of HBV- and HBV+ patients with each other (p<0.05, R>0.05, positive correlation in orange, negative correlation in blue). (H) Heatmap of correlation between genus level taxon 1 and taxon 2 in tumor tissues of HBV- and HBV+ patients with each other.
